# The Challenges and Opportunities of LncRNAs in Ovarian Cancer Research and Clinical Use

**DOI:** 10.3390/cancers12041020

**Published:** 2020-04-21

**Authors:** Martín Salamini-Montemurri, Mónica Lamas-Maceiras, Aida Barreiro-Alonso, Ángel Vizoso-Vázquez, Esther Rodríguez-Belmonte, María Quindós-Varela, María Esperanza Cerdán

**Affiliations:** 1 EXPRELA Group, Centro de Investigacións Científicas Avanzadas (CICA), Departamento de Bioloxía, Facultade de Ciencias, INIBIC-Universidade da Coruña, Campus de A Coruña, 15071 A Coruña, Spain; martin.salamini.montemurri@udc.es (M.S.-M.); monica.lamas@udc.es (M.L.-M.); aida.barreiro@udc.es (A.B.-A.); esther.belmonte@udc.es (E.R.-B.); 2 Translational Cancer Research Group, Instituto de Investigación Biomédica de A Coruña (INIBIC), Carretera del Pasaje s/n, 15006 A Coruña, Spain; maria.quindos.varela@sergas.es

**Keywords:** diagnosis, prognosis, therapy, molecular mechanisms, bioinformatics tools

## Abstract

Ovarian cancer is one of the most lethal gynecological malignancies worldwide because it tends to be detected late, when the disease has already spread, and prognosis is poor. In this review we aim to highlight the importance of long non-coding RNAs (lncRNAs) in diagnosis, prognosis and treatment choice, to make progress towards increasingly personalized medicine in this malignancy. We review the effects of lncRNAs associated with ovarian cancer in the context of cancer hallmarks. We also discuss the molecular mechanisms by which lncRNAs become involved in cellular physiology; the onset, development and progression of ovarian cancer; and lncRNAs’ regulatory mechanisms at the transcriptional, post-transcriptional and post-translational stages of gene expression. Finally, we compile a series of online resources useful for the study of lncRNAs, especially in the context of ovarian cancer. Future work required in the field is also discussed along with some concluding remarks.

## 1. Introduction

Ovarian cancer (OC) is the second most common cause of death worldwide due to gynecological cancers. There were some 295,000 new cases and 185,000 deaths around the globe in 2018, with increasing trends predicted [1]. According to the type of cell in which the tumor originates, OC can be classified as stromal, germinal or epithelial (EOC), the latter being the most common, accounting for 90% of cases. Within EOC, five histological subtypes can be distinguished: high-grade serous, low-grade serous, mucinous, clear-cell and endometrioid. They are distinguished on the basis of histological structure; mutations in certain proto-oncogenes or tumor suppressor genes; chemosensitivity; spreading behavior; and the most worrisome, prognosis. Little is known about the etiology of the disease, although some genetic and environmental risk factors have been identified; e.g., BRCA1/2 mutations, and low or null parity, respectively. OC, and EOC patients particularly, are usually diagnosed at an advanced stage of the disease owing to the asymptomatic character of the tumor during its onset and initial development, leading to a five-year overall survival rate below 40% [2]. In contrast, early diagnosis correlates with a much better prognosis. Unfortunately, no early biomarkers have been approved for clinical use so far, although some attempts have been made using multivariate index assays, such as the risk of ovarian malignancy algorithm (ROMA) or Overa [3]. The first-line treatment of OC consists of surgical resection of the tumor and administration of platinum derivatives, taxanes and/or bevacizumab, which is a monoclonal antibody targeting vascular endothelial cell growth factor (VEGF). Additionally, olaparib, which is an inhibitor of poly (ADP-ribose) polymerase (PARPi), was authorized as first-line maintenance treatment for BRCA-mutated high-grade serous OC patients who have shown complete or partial response to platinum. Despite the benefits of these therapies, most patients experience relapses and the tumor even becomes resistant to the treatment. As a second-line treatment, PARPi, doxorubicin or gemcitabine are administered [4].

**Figure 1:**
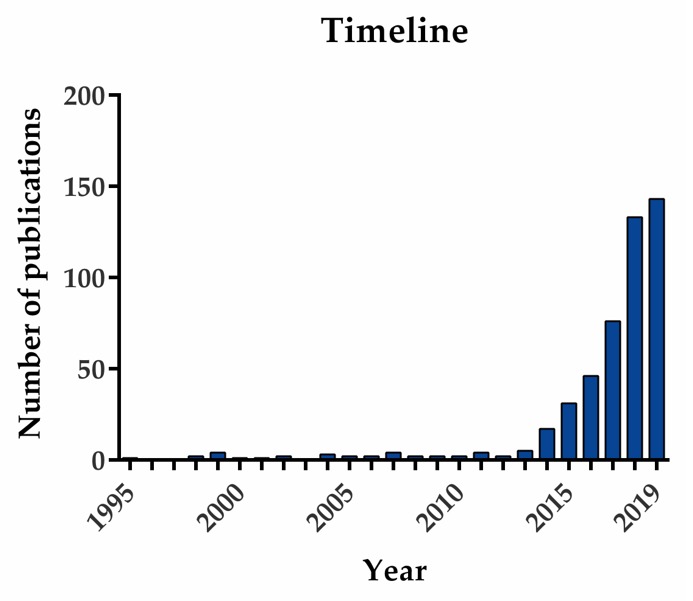
Timeline of released publications containing “LncRNA AND ovarian cancer” in PubMed online library.

Long non-coding RNAs (lncRNAs) are transcripts that were identified in genomic studies during the late 1990s and 2000s. They are defined as longer than 200 nucleotides and are presumed not to encode proteins. The peculiarity of lncRNAs is their ability to regulate gene expression at many different levels, by modulating chromatin remodeling, transcription, and alternative splicing, and generating micro RNAs (miRNAs) or producing short biologically active peptides [5]. They actively participate in all the events involved in tumor development and spread, and even in treatment resistance in bladder cancer, colorectal cancer, multiple myeloma and others, including OC [6]. Additionally, their expression is tissue-specific [7] and they can be detected in fluids, making them potential biomarkers [8]. LncRNAs have not been as deeply studied as their counterparts, miRNAs, and many questions remain about their mechanisms of action and effects in the context of cancer, including OC. Interestingly, the number of different lncRNAs associated with OC, and consequently publications on the topic, has recently grown exponentially (Figure 1), making previous reviews out-of-date owing to the overwhelming mass of data being produced. The aim of this review is to update and compile information about the lncRNAs related to OC; their importance in the clinical setting as diagnostic and prognostic tools; and their relationship to cancer hallmarks and their molecular mechanisms of action in the cell. In addition, we briefly comment on the online bioinformatics tools used in the reviewed papers. Finally, future work required in the field is also discussed along with some concluding remarks.

## 2. Clinical Relevance of LncRNA in OC: Diagnosis, Prognosis and Treatment Resistance

As mentioned above, there are currently no efficient and routine methods for early diagnosis of OC. Only in a minority of cases, wherein the condition is suspected on the basis of either unspecific symptoms or familial antecedents, can preventive measures be taken, such as gynecological explorations; imaging techniques, such as transvaginal sonography; or blood tests to measure cancer antigen 125 (CA-125) together with other proteins with informative value, such as transferrin or HE4 [3]. If a tumor is detected, further and more sophisticated explorations are conducted: imaging techniques such as computed tomography (CT) or positron emission tomography (PET), and laparotomy or surgery to: (i) extract a sample for biopsy/histology-based diagnosis, (ii) study the extent of the disease (localized or generalized-staging) and/or (iii) proceed with therapeutic surgical debulking/cytoreductive surgery of the tumor [4]. These techniques are either nonspecific or only valid for late detection [3].

In the process of developing new, specific, differential molecular markers for OC, lncRNAs are proposed as a new generation of clinical tools. They have been studied in OC patient samples using reverse transcription quantitative polymerase chain reaction (RTqPCR), microarray hybridization and (fluorescence) in situ hybridization ((F)ISH), mainly from cancerous tissue, and compared with adjacent normal tissue or samples from healthy patients. Usually, their levels differ from those in the controls (Table S1), and such aberrant lncRNA expression also correlates with clinicopathological parameters, such as histological type, tumor size or metastasis (Table 1).

The presence of several lncRNAs has been correlated with epithelial subtypes. For instance, Casc2 and FLJ33360, respectively, distinguish the serous and high-grade serous subtypes from the others [17,66]; SNHG15 differentiates low-grade serous, mucinous, endometrioid and clear cell from high-grade serous, endometrioid and non-differentiated [67]. The expression of many lncRNAs associated with OC, namely, ANRIL, CCAT1, CCAT2, EIBC, GAS5, HOXA11-AS, lncARSR, lncBRM, Lnc-OC1, MIR4697HG, NEAT1, SOX2OT [6] and SOCAR [64], also correlate with blood levels of CA-125, which could improve the patient’s follow-up, since lncRNAs are highly cancer-specific [7].

The spreading of OC in patients is classified into four stages according to the International Federation of Gynecology and Obstetrics (FIGO), which in addition to tumor size and histological grade/cell-dedifferentiation correlates negatively with the prognosis and survival of patients [68]. LncRNA expression correlates with FIGO staging (Table S1), either grouping I–II and III–IV as in ABHD11-AS1 [10], or discerning each of the four stages precisely, as reported for AGAP2-AS1 [69]. In this review, we will focus on the clinicopathological variables associated specifically with each of the FIGO stages. FIGO stage I means the tumor is limited to the ovaries; the main studied variable that affects this stage is primary tumor size. The bigger the tumor, the more developed it is and the poorer the prognosis, although this criterion could be misleading because some tumors are benign. The histological grade, that is, the state of tumor cell dedifferentiation, relates to the tendency of the tumor to invade surrounding tissues and organs. It is associated with FIGO stage II and with the malignancy and aggressiveness of the tumor. The next phase of tumor progression is the acquisition of invasive capacity, which finally determines how close the tumor can get to adjacent tissues or vessels. Both GAS5 [22] and MLK7-AS1 [40] correlate with the depth of tumor invasion. Once the tumor cells reach vessels, they usually invade lymph nodes first (FIGO stage III) and the peritoneal cavity because of its proximity, but eventually other organs are reached (FIGO stage IV). Correlations between FIGO stages and the associated clinicopathological features are not always perfect, perhaps because of experimental variability.

Besides their diagnostic value, individual lncRNAs can predict the survival of OC patients according to their levels in the cohorts studied, yielding different survival outcomes between patients with high and low lncRNA expression levels. As seen in Table 2, most of them correlate with patients’ overall survival (OS), whereas fewer correlate with progression-free survival (PFS) or disease-free survival (DFS) periods, as shown in Table 3.

LncRNAs are clinically important in the context of OC in another sense because they are known to be active in developing resistance to treatments (Table 4), leading to relapse and poor patient prognosis. Most of these have been associated with resistance to chemotherapeutics. The most widespread clinical treatments are platinum salts such as cisplatin or carboplatin, and taxanes such as paclitaxel [4]. Depending on their specific roles, lncRNAs can either promote or reduce resistance to the treatment, as summarized in Table 4.

Some have been implicated in resistance to experimental drugs such as bufalin and physcion 8-O-B-glucopyranoside; for example, NORAD [115,116]. Some, such as CTD-2589M5.4, which is co-expressed with some ATP-binding cassette (ABC) genes, are only indirectly related to chemoresistance [102]. One, FAM83H-AS, has even been identified as conferring radioresistance in vitro [15].

LncRNAs can also help to stratify patients for treatment choice in order to increase responsiveness and efficacy. This is the case for SNHG5 in reference to paclitaxel [62], and for RP11-135L22.1 [48] and LINC00515 [56] regarding platinum drugs. The expression of these lncRNAs differs between responder and non-responder patients, which enables their stratification. NEAT1 is the only lncRNA identified so far as being involved in resistance to olaparib (PARPi) treatment, which is a targeted therapy against BRCA1/2-mutated tumors [105].

Despite great efforts to use these advances in the clinic, only six of the lncRNAs listed in Table S1 have been identified in OC patients’ blood serum samples; namely, E2F4AS [117], FEZF1-AS1 [20], FLVCR1-AS1 [41], LINK-A [26], MLK7-AS1 [40] and aHIF [13], the last-named being isolated specifically from exosomes in the serum. MEG3 and MALAT1 are probably on the way to being added to the list since they have been identified in exosomes derived from OC cell lines [26,107]. Interestingly, the serum and tumor tissue levels of aHIF, FEZF1-AS1, FLVCR1-AS1, LINK-A, and MLK7-AS1 correlate positively, providing a proof of concept for the usefulness of lncRNAs in liquid biopsy. Such is the interest caused by these findings that there are currently two ongoing clinical trials on lncRNAs for OC, one aiming to search lncRNAs in exosomes from patients [118], and the other to discover new lncRNAs implicated in OC by a multi-omic approach [119].

## 3. LncRNAs Implicated in OC Development and Progression

The study of lncRNAs is an emerging field. According to GENCODE Release 33 [120], the number of lncRNA genes known at the time of submission of this review was 17,952 (16,892 already confirmed and 1060 to be experimentally confirmed). Only a small portion has been studied in detail; and of these, to the best of our knowledge, just 215 have been proven to be involved in OC so far (Table S1).

Long non-coding RNAs, as key regulators of gene expression at different levels, take part in many physiological processes. Their dysregulation can, therefore, contribute to the aberrant functioning of the cell leading to different diseases, including cancer [6,121].

**Figure 2:**
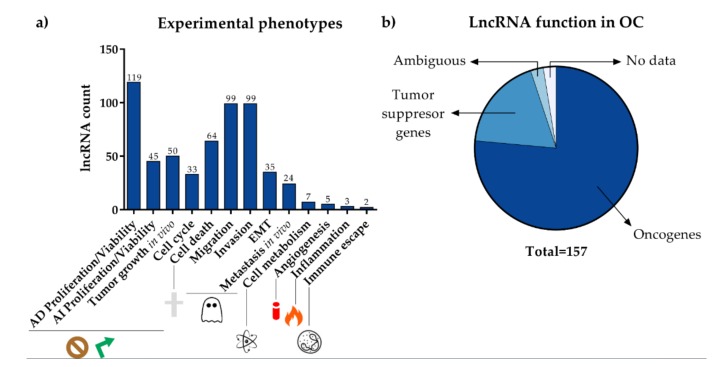
Summary of the functional implications of lncRNAs based on experimental evidence. (**a**) Number of lncRNAs contributing to the indicated phenotype or cancer hallmark; (**b**) Proportion of lncRNAs according to their net effect on ovarian cancer onset, development, and progression. AI: anchor-independent; AD: anchor-dependent.

Although a few of them are best known/studied in the OC context, e.g., MALAT1, HOTAIR, H19, XIST, UCA1, PVT1, GAS5 and MEG3, 157 different lncRNAs have been shown experimentally to be associated with OC (Table S1). Of these, around 50%, 81, have not been cited in previous reviews but participate actively in OC. Most experiments so far reported are based on the knockdown or overexpression of specific lncRNAs in different cancer cell lines and assessments of their corresponding cancer-related phenotypes in culture (in vitro) or with xenografts (in vivo) (Figure 2a). Surveying the function of lncRNAs globally (Figure 2b), 120 are considered as oncogenes, 114 confirmed and six putative; and 29 are considered as tumor suppressor genes, 25 confirmed and four putative. SPRY-IT1 [26], MEG3 [122,123], XIST [54,99] and TTN-AS1 [65,124] statuses remain unclear because the shreds of evidence collected are ambiguous. The experimental data about TC0101686, TC0100223, TC0901107, and TC1500845 show only that they are transcriptionally activated by estrogens. No phenotypic consequence of dysregulation of these latter four lncRNAs has been reported [42].

LncRNAs are involved in proliferation, tumor growth in vivo, cell cycle control, cell death, migration, invasion, epithelial-to-mesenchymal transition (EMT), metastasis in vivo, angiogenesis and cell metabolism, all related to the hallmarks of cancer [125]. The only two hallmarks of cancer that have not yet been related to lncRNA in the context of OC are “genomic stability and mutation” and “enabling replicative immortality.”

The most studied characteristic, and also the most frequently affected by changes in lncRNA expression, accounting up to 119 different lncRNAs, is anchorage-dependent cell proliferation, which determines cell division rate and viability. Closely related to this, and affected by fewer lncRNAs, is anchorage-independent cell proliferation, which measures the ability of cells to survive and proliferate in the absence of a solid support, a characteristic of malignant cells. After mouse xenografting, lncRNAs have also been shown to influence the size and weight of the tumor, some replicating the same scenario as in patients, such as AB073614 [9], EPB41L4A-AS2 [23], GAS5 [22], KCNQ1OT1 [35], LINC00565 [37], TINCR [51] and TPT1-AS1 [53].

Loss of cell cycle control is also a consequence of lncRNA dysregulation, as in the case for MNX1-AS1 [6] and SPRY4-IT1 [26], described previously; and KB-1471A8.2 [103] and Casc15 [12], which were recently shown to be relevant in this context. This effect is exerted by ultimately controlling the expression of cyclins, cyclin-dependent kinases and cyclin-dependent kinases inhibitors, influencing G_0_ arrest or cell cycle halting. These features are related to the cancer hallmarks of proliferative signal maintenance and evasion of growth suppressors, described by Hanahan and Weinberg.

Avoidance of cell death, causing cell immortalization, is another important hallmark of cancer. The most studied type of cell death is apoptosis, regulated by lncRNAs such as HAL [126] and LncRNA-ATB [127] among others. However, GAS5 can trigger the formation of the inflammasome, leading to pyroptosis, a highly inflammatory type of programmed cell death [128]. In this regard, some lncRNAs have been proven to induce the autophagic response, as with MEG3 [6] and MALAT1 [107], or inhibit it, as with HOTAIR [26], HULC [6] and RP11-135L22.1 [48]. Importantly, these groupings do not coincide with oncogene or tumor suppressor status; the net effect of autophagy in the context of cancer has been questioned because its outcomes are ambiguous [129].

Migration and invasion in vitro are, after AD proliferation, the characteristics most commonly influenced by lncRNAs, representing motility and the ability to degrade the extracellular matrix. Many of the lncRNAs described in OC (Table S1) can affect the EMT process, as demonstrated through molecular markers such as Snail, Slug, E-cadherin, N-cadherin, and Vimentin [130]. Finally, in vivo metastasis is also influenced by lncRNAs, some of which recapitulate patients’ fate and/or tumor behavior, as in the case of DANCR [11,131], FAM83H-AS1 [15], MAGI1-IT1 [34], MLK7-AS1 [40] and TPT1-AS1 [53]. These four features (migration, invasion, EMT and in vivo metastasis) are associated with the hallmark “invasion and metastasis.”

Interestingly, although this effect has been less studied, some lncRNAs control cell metabolism by regulating key enzymes in metabolic pathways, contributing mainly to stimulating glycolysis instead of aerobic metabolism (the Warburg effect). In the glycolysis pathway from glucose to pyruvate, the expression of hexokinase 2, catalyzing the first step, is activated by LINC00504 [70]; NRCP activates the second enzyme in the pathway, glucose-6-phosphate isomerase [26]; after that, 6-phosphofructo-2-kinase/fructose-2,6-biphosphatase 2 is induced by LINC00092 [31]; in the fourth step catalyzed by aldolase, two forms of the enzyme, A and C, are activated by NRCP [26]. The final step is catalyzed by pyruvate kinase isozyme M2, which is activated by both LINC00504 and H19 [70,132]. Moreover, LINC00504 activates the expression of pyruvate dehydrogenase kinase 1 (PDK1), which inhibits pyruvate dehydrogenase by phosphorylation [70], thereby inhibiting the Krebs cycle. SNHG3 is known to affect metabolism but its net effect on metabolic switching is not clear since it activates key enzymes in glycolysis such as pyruvate kinase, but also Krebs cycle enzymes such as pyruvate dehydrogenase and isocitrate dehydrogenase, and oxidative phosphorylation components, such as ubiquinol–cytochrome c reductase hinge protein [88]. Less directly, GHET1 [24] and LINK-A [26] are also related to metabolic switching, since they regulate the expression of HIF1a, which is also responsible for inducing the Warburg effect.

Angiogenesis is a driving force for tumors owing to increased vascularization, which guarantees nutrient delivery for tumor growth and provides a route through which malignant cells can colonize other tissues. Although angiogenesis is a hallmark of cancer and an important therapeutic target, only a few published studies have addressed the role of lncRNAs in it. It has been established that MALAT1 promotes angiogenesis by inducing VEGF and fibroblast growth factor expression in OC [105], but recently two others have also been shown to have pro-angiogenic properties, named DANCR [133] and HNF1A-AS1 [134]. They activate the expression of VEGF and SEMA4D, respectively, which are powerful inducers of blood vessel formation. Since HIF1a, which is regulated by GHET1 and LINK-A, controls VEGF expression, these two lncRNAs are also related to angiogenesis.

A few lncRNAs, such as AC104699.1.1 and RP11-284N8.3.1, which are related to activation of the tumor microenvironment, are also involved in promoting inflammation [26]. Interestingly, GAS5 can activate pyroptosis, which, as mentioned in the cell death paragraph, triggers an excessive inflammatory response, collaborating with tumor progression [128].

Only one lncRNA, HOTTIP, has been cited in reference to the avoidance of immune surveillance. Others, such as CTD-20202K17.1, are indirectly related to this cancer hallmark. HOTTIP increases the expression and secretion of IL-6 by tumor cells, which triggers PD-L1 expression through the STAT3 pathway in neutrophils [87]. CTD-2020K17.1 controls CARD11 expression, which is proposed to modulate tumor immune-surveillance [135].

## 4. Regulatory Molecular Mechanisms of LncRNAs in OC

**Figure 3:**
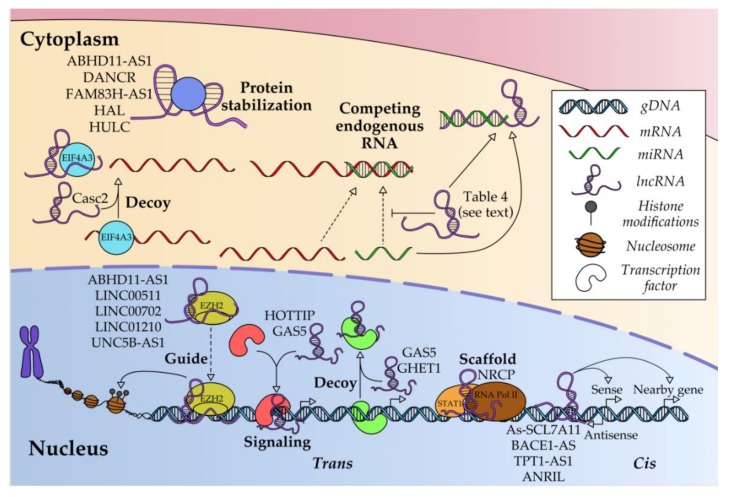
Scheme of different mechanisms by which lncRNAs control the expression of target genes in OC.

The phenotypes described are a consequence of target gene regulation by lncRNAs by different mechanisms, affecting gene expression at the transcriptional, post-transcriptional and post-translational levels in OC (Figure 3).

### 4.1. Transcriptional Regulation

LncRNAs can regulate transcription in two different ways according to their locations of action in relation to their own transcription sites. Their effects can be exerted either at the same or a nearby locus, i.e., in cis, or at distant sites on the same or different chromosomes, i.e., in trans.

LncRNAs can associate with transcriptional protein complexes to modulate their function, changing their capacity to bind DNA and modulate the transcription of target genes via signaling, guide, decoy and/or scaffold mechanisms. By the signaling mechanism, lncRNAs participate in transcriptional machinery or transcription factor (TF) recruitment to the promoter of their downstream-regulated genes. They either promote transcription by facilitating RNA polymerase binding, as in the case of HOTTIP, which binds to c-Jun to promote IL-6 transcription [87]; or, in contrast, repress transcription, as in the case of GAS5, which brings E2F4 to the PARP1 promoter region, precluding RNA polymerase binding [46].

In another described mechanism, lncRNAs guide specific epigenetic repressors such as polycomb repressive complexes (PRC) to the locus of a certain gene by a mechanism based on DNA-RNA complementarity, leading to histone modification and therefore to gene silencing. This kind of regulation can be also in cis, although no examples have yet been described for OC. However, several examples have been described in OC for lncRNA-based PRC2 guidance, specifically of EZH2, an active subunit of PRC, to the loci of target genes: LINC00511 and TP73-AS1 guide EZH2 to the p21 locus [72,95]; LINC00702 guides it to the KLF2 locus [39]; LINC01210 to the KLF4 locus [27]; ABHD11-AS1 to the TIMP2 locus [136]; and UNC5B-AS1 to the NDRG2 locus [137].

LncRNAs can also act as decoys to sequester TFs, disabling their interaction with their target gene promoters, like “protein sponges”; i.e., decreasing their affinity with DNA. GAS5 acts as a decoy with the activator CEBPB, precluding its interaction with the GDF15 promoter, thereby inhibiting its transcription [138]. Similarly, GHET1 prevents the binding of VHL to the HIF-1 promoter [24].

LncRNAs can also act as a scaffold by interacting with two or more proteins simultaneously, bringing them together and stabilizing the complex formed. For instance, lncRNA NRCP joins STAT1, which is a TF implicated in the JAK/STAT signaling pathway, and RNA Pol II [26].

These categories are not mutually exclusive. For instance, Linc00176 acts as a scaffold to keep BCL3 and p50 together (NF-κB family) and at the same time recruits this complex to the ceruloplasmin promoter to activate its expression [139]. Some reports of transcriptional regulation by lncRNAs do not fit this classification at all, as in the case of FAL1, which increases the stability of BMI (another subunit of PRC), thereby allowing epigenetic marking of the p21 gene to be silenced [31,42].

Apart from trans regulation, lncRNAs can affect the transcription of neighboring genes. They usually regulate in cis the transcription of a sense gene that normally encodes a protein; this is the case for As-SLC7A11, which negatively regulates SLC7A11 [6], or BACE1-AS and TPT1-AS1, which positively regulate BACE1 [21] and TPT1 [53]. They can also transcriptionally regulate nearby genes, as in the case of ANRIL, the expression of which correlates positively with the master tumor suppressors p14-ARF and p15-CDKN2B in normal tissues, and much more positively and statistically significantly with p14-ARF, p16-CDKN2A and p15-CDKN2B in tumor tissues [140]. Indirect evidence shows that the lncRNAs ADAMTS9-AS1, RP11-597D13.9, FAS-AS1, AC093818.1, AK130076, GTSE1-AS1, and RP11-199F11.2 could be associated with ADAMTS9, FAM138B, FAS, PDK1, PTEN, GTSE1, and TP53 coding genes, respectively, all of them being implicated in OC [14,42].

### 4.2. Post-Transcriptional Regulation

The most frequently identified regulatory mechanism of action of lncRNAs is acting as competing endogenous RNAs (ceRNA) with miRNAs, which regulate the fate of mRNAs that encode proteins involved in cancer. These regulatory relationships among ncRNAs are not mutually exclusive, and several lncRNAs can act on the same miRNA targets, such as ANRIL, H19, and HOST2 that sequester let-7a, which regulates the expression of HMGA2, c-Myc, IGF2BP, Dicer and IMP3; and HOTAIR, lncARSR, and MALAT1 that sequester miR-200c, which modulates the expression of EMT regulators such as ZEB1/2 or Snail. Table 5 presents the numerous lncRNA/miRNA/mRNA axes so far known to be involved in OC. Another mode of post-transcriptional regulation is the sequestering of RNA processing factors. LncRNA Casc2 sequesters (decoys) EIF4A3, which takes part in splicing and is associated with monitoring mRNA quality before translation is initiated, thereby preventing the translation of proteins involved in the NF-κB, PI3K, and AKT signaling pathways [141].

### 4.3. Post-Translational Regulation

According to bioinformatics predictions, SNHG3 could interact with EIF4IIIA, a factor involved in regulating protein translation [88]. Otherwise, no lncRNAs have been described as significant in regulating protein translation in the context of OC. However, they can have important post-translational effects by modulating the half-lives of the proteins they bind by stabilizing them, as in the case of ABHD11-AS1 and RhoC [10]; DANCR and UPF1 [11]; and FAM83H-AS1 and HuR [15]. UCA1 can act as a scaffold between two proteins; e.g., for cytoplasmic Yes-associated protein (YAP) and angiomotin, it promotes YAP dephosphorylation and its translocation to the nucleus to act as a transcription coactivator [157]. Other examples of post-translational regulation are the lncRNA HAL, which interacts with Twist1, this interaction being important for promoting EMT [126], and the lncRNA HULC, which interacts with ATG7 and inhibits the autophagy pathway [6].

We can find examples in which one lncRNA belongs to more than one of these categories according to its molecular mechanisms of action. For instance, the lncRNA NEAT1 can bind/sequester at least four different miRNAs: miR-194, miR-382-3p, miR-506 and miR-124-3p [6,21,105]. Another example is the lncRNA miR503HG, which regulates miR-31-5p, surprisingly at both the transcriptional level by promoting the methylation of its promoter region, and post-transcriptionally by sequestering it, thereby preventing its union with the target mRNAs [100]. Similarly, PVT1 guides EZH2 to the miR-214 promoter and simultaneously sequesters miR-133a [26] and miR-140 [74]. NCK1-AS1 binds to c-Cbl to protect it from ubiquitination, thereby precluding proteasomal degradation, and it also regulates its own sense transcript by sequestering miR-137, which binds to NCK1 mRNA [114].

### 4.4. Regulation of LncRNA Expression

Due to their ability to modulate the behavior of OC cells, the regulation of lncRNAs in itself is a matter for study, and some research has been done on this topic.

Some physiological conditions can trigger the transcription of certain lncRNAs, such as aHIF [158], which is induced under hypoxic conditions like those existing in the tumor microenvironment. Several lncRNAs are modulated by, e.g., estrogens in the case of TC0100223, TC0101441, TC0101686, TC0901107 and TC1500845 [42]; and also by some experimental drugs, as Casc2 is promoted by sanguinarine; anisomycin activates BACE1-AS [21] and MEG3 [123]; sinomenine hydrochloride inhibits HOST2 [83]; actinomycin D promotes MEG3 transcription [42]; or valproic acid inhibits H19 [107]. For lncRNAs considered as tumor suppressor genes, hypermethylation of the coding gene promoter can cause downregulation, and hence OC, as with ZNF300P1, which was found to be hypermethylated in about 80% of OC cases [42]. Demethylating agents such as 5’-AZA-2’ or curcumin effectively restore the expression of lncRNAs such as MEG3 [26] or MAGI2-AS3 [149].

There are examples in which the TFs responsible for lncRNA transcription have been detected. Thus, CREB1 activates HAS-AS2 transcription [79]; FOXO4 activates PVT1 transcription [74]; Nkx3-1, HNF-1, aMEF-2 and MEF-2A are TFs take part in the transcriptional regulation of LOC100190986 [21]. Some signaling pathways that trigger lncRNA transcription have also been identified; for instance, CXCL14 produced by surrounding fibroblasts triggers LINC00092 transcription [31]; TGF-β1 activates MALAT1 [21], PTAR [154] and PTAL [153] transcription; or pEGFR activates ABHD11-AS1 through STAT3 [136]. The lncRNA UCA1 has been described as being activated by a super-enhancer [157].

The regulatory network becomes more complicated when lncRNAs regulate each other, as in the cases of MEG3 regulated by AGAP2-AS1 [69]; NEAT1 regulated by LSINCT5 [21]; or the tight relationship between Lnc-SOX4-1 and PVT1 [42]. A regulatory feedback loop has been discovered between Casc15 and miR-221 [12]. Finally, lncRNAs can be stabilized by post-transcriptional modifications, such as RHPN1-AS1 methylation [77], or by proteins, such as HuR, which binds NEAT1 [6], at the extent of OC.

## 5. Bioinformatics Resources for LncRNA Research

The aim in this final section is to review the online tools available for lncRNA research and used in many of the articles compiled in this review in reference to OC. These web-based resources consist of the databases shown in Table 6, which allow researchers to gain access to a vast amount of information in an easy and localized way, and/or the prediction tools shown in Table 7, which are mostly user-friendly, avoid the constraints of bioinformatics programming and do not require expert or advanced knowledge.

One of the most used databases is GEPIA [159], which offers customizable analysis of expression levels in tumors and normal samples using the RNA-sequencing data obtained from The Cancer Genome Atlas (TCGA) and Genotype-Tissue Expression Portal, respectively. Similarly, Cancer Cell Line Encyclopedia (CCLE) [160] collects information, including details of lncRNAs, about gene expression, mutations and methylation status in more than a thousand cancer cell lines. TANRIC [161] combines information from TCGA, CCLE and independent datasets, focusing on lncRNA expression profiles in an interactive display.

Since the study of lncRNAs is an emerging but growing field, and some lncRNAs can be functionally identified as new transcripts, a consensus for indexing the different aliases needs to be established, a mission covered by several databases, such as LNCipedia [162], NONCODE [163], AnnoLnc [164], lncRNAdb [165] or lncRBase [166], containing annotations and gene information.

There are also databases that record the already-described interactions between lncRNAs and DNA, other RNAs and proteins in different contexts, enabling new mechanisms of action to be discovered. For instance, RNAInter [167] and StarBase-ENCORI [168] contain a vast, multi-omic-based amount of information about whole RNA interactomes, whereas miRTarBase [169] is only valid for miRNA interactions. In other databases, information about how lncRNAs are regulated can be retrieved, as in the case of ORTI [170], which contains information relating to TFs and their regulated genes, including lncRNAs. One reason why gene expression can be dysregulated is methylation status. This can be looked up easily in DiseaseMeth [171], which collects aberrant human methylomes; or more specifically for this topic, in Lnc2Meth [172], which includes regulatory relationships between human long non-coding RNAs and DNA methylation.

Two databases have mainly experimental purposes; namely, LncRNA2Target [173] and CRISPRlnc [174]. The former collects lncRNA knockdown or overexpression experiments in human and mouse to check target genes by immunoprecipitation, RNA pull-down, immunofluorescence, microarray hybridization or RNA-seq techniques. The latter contains a curated database with experimental evidence of single guide RNAs to perform knockouts of lncRNAs in different species.

Finally, there are several databases for specific relationships between lncRNAs and diseases, such as LncTarD [176] and LncRNA disease [177]; or, more specifically in the context of cancer, LncMAP [178], Lnc2Cancer [179], CRlncRNA [180], EMT-Regulome [181] and RHPCG [182].

Apart from databases, there are useful tools for predicting the behavior of lncRNAs in terms of molecular interactions. Some of these can predict interactions with proteins, such as RPISeq [183], RPI-Pred [184], lncPro [185] and catRAPID [186]. Others predict the probability of interaction with other RNAs, such as mirDIP [187] and LncRRISearch [188]. There are also tools that can predict the regions where lncRNAs bind to genomic DNA, named LongTarget and LongMan [189], the first being for a localized genomic region and the second for larger genomic areas. Finally, lncRNA secondary structure is important for these interactions, and it can be predicted by the RNAfold web server [190], which calculates the minimum free energy conformation.

## 6. Conclusions and Perspectives

LncRNAs are emerging tools important for diagnosis, prognosis and therapy. We have extensively reviewed the latest advances in lncRNA research in the context of ovarian cancer (OC). Although we can verify that there is a vast list of lncRNAs associated with OC, the problem arises when it comes to finding out which ones are the most useful to improve the management of the disease. As has been shown in this review and can be seen in the data provided in the Supplementary Materials, some lncRNAs have been clearly identified as oncogenes, and alterations in their expression and their consequences in OC have been verified in independent cohorts, even by independent laboratories.

There are three main characteristics required to use these molecules as useful and valid biomarkers in OC diagnosis. The first one is that their variations in expression could be considered OC/cancer-specific. In practice, many of the reviewed lncRNAs have been tested by the receiver operating characteristic analysis to be able to successfully discern between OC and normal patients. The second is that they should occur in the early stages of the disease, although, only a few studies take into account samples from benign or borderline tumors which represent the earliest manifestation of the disease. The last required feature is that simple methods could be established for their quantification and detection, which allow their identification for massively testing the population at risk. However, there are pitfalls for implementing lncRNA screening in clinical practice because of technical issues and lack of ease of use and readiness. To detect lncRNAs, RNA-seq, RTqPCR, microarray hybridization and (F)ISH are the most common research techniques, but they are time-consuming. With the shifting perspectives in this field, new techniques are arising, such as loop-mediated isothermal amplification [191] and isothermal reverse transcription-recombinase polymerase amplification [192], which should make this task much easier and more feasible.

As we have seen throughout the review, some lncRNAs have also been related to sensitivity/resistance against drugs used in the treatment of OC or with more aggressive forms of the disease. Consequently, they could be used in prognosis and decision-making about the most suitable protocols in first and second-line therapies. Once the scientific community has identified and reached a consensus on which lncRNAs are the most useful in OC diagnosis and/or prognosis; their easy screening will be a scientific and technological advance that may be implemented through liquid biopsies from patient serum or different body fluids.

According to the evidence provided about the molecular mechanisms of some of these reviewed lncRNAs, they could be druggable targets for small interfering RNAs or antisense oligonucleotides, but this therapy is not yet possible in clinical practice owing to delivery problems, which could be solved by ongoing nanotechnological approaches. Additionally, in a recently opened research line, lncRNAs were regarded as true coding RNAs producing small peptides of less than 100 amino acids, which could be the real effectors of the lncRNAs in the cells and could have profound implications for cancer physiology [193]. These lncRNAs and their derived peptides, which are also putative therapeutic targets to be tested in the next years, can be consulted in the database SmProt [175].

Other upcoming approaches in this field could involve developing animal models for knockout or overexpression of lncRNAs, or patient-derived xenografts for personalized therapy. Promising data for better patient care awaits us as a result of ongoing and future research in this field.

## Figures and Tables

**Table 1:** Long non-coding RNAs (lncRNAs) associated with clinicopathological variables in ovarian cancer (OC) patients.

Tumor Size		Histological Dedifferentiation Grade		Lymph Node Metastasis		Distant Metastasis	
LncRNA	Ref.	LncRNA	Ref.	LncRNA	Ref.	LncRNA	Ref.
AB073614	[9]	ABHD11-AS1	[10]	AB073614	[9]	DANCR	[11]
Casc15	[12]	aHIF	[13]	AC093818.1	[14]	FAM83H-AS1	[15,16]
Casc2	[17]	ANRIL	[6,18]	ADAMTS9-AS2	[19]	FEZF1-AS1	[20]
DUXAP10	[6]	ASAP1-IT1	[21]	AK130076	[14]	GAS5	[22]
EPB41L4A-AS2	[23]	Casc15	[12]	ANRIL	[6]	GHET1	[24]
FAM83H-AS1	[15]	Casc2	[17]	Casc2	[17]	JPX	[25]
GAS5	[22,26]	CCAT1	[6]	CCAT1	[6]	LINC01210	[27]
GHET1	[24]	CCAT2	[6]	CCAT2	[6]	LncSOX4	[28]
HAGLROS	[29]	CPS1-IT1	[6]	CPS1-IT1	[6]	LUCAT1	[30]
HOTAIR	[31]	DLEU1	[32]	DLX6-AS1	[33]	MAGI1-IT1	[34]
JPX	[25]	DUXAP10	[6]	EIBC	[6]	MALAT1	[6]
KCNQ1OT1	[35]	EIBC	[6]	FAM83H-AS1	[15]	MCM3AP-AS1	[36]
LINC00565	[37]	EPB41L4A-AS2	[23]	FAS-AS1	[14]	MEG3	[38]
LINC00702	[39]	FAM215A	[21]	FEZF1-AS1	[20]	MLK7-AS1	[40]
LncSOX4	[28]	FAM83H-AS1	[16]	FLVCR1-AS1	[41]	NEAT1	[42]
MORT	[43]	FEZF1-AS1	[20]	GAS5	[6]	PCAT6	[44]
PCAT-1	[45]	GAS5	[6,46]	GTSE1-AS1	[14]	SNHG12	[47]
RP11-135L22.1	[48]	HOTAIR	[21]	HMMR-AS1	[49]	SNHG20	[50]
TINCR	[51]	HOXA11-AS	[42]	HOTTIP	[52]	TPT1-AS1	[53]
TPT1-AS1	[53]	KCNQ1OT1	[35]	HOXD-AS1	[6]	XIST	[54]
		LINC00339	[55]	LINC00515	[56]		
		LINC00472	[42]	Linc-ROR	[57]		
		LINC01088	[58]	lncARSR	[6]		
		Linc-ROR	[59]	lncBRM	[6]		
		lncARSR	[6]	Lnc-OC1	[6]		
		lncBRM	[6]	MCM3AP-AS1	[36]		
		Lnc-OC1	[6]	MIR22HG	[6]		
		MEG3	[38]	MLK7-AS1	[40]		
		MIR22HG	[6]	MNX1-AS1	[6]		
		MNX1-AS1	[6]	NBAT-1	[6]		
		NBAT-1	[6]	NEAT1	[6]		
		NEAT1	[31]	NONHSAT076754	[60]		
		NONHSAT076754	[60]	PCAT6	[44]		
		SNHG20	[61]	RP11-199F11.2	[14]		
		SNHG5	[62]	SNHG20	[61]		
		SOX2OT	[6]	SNHG3	[63]		
		SPRY4-IT1	[6]	SNHG5	[62]		
		TC0100223	[21]	SOCAR	[64]		
		TC0101441	[21]	SOX2OT	[6]		
		TPT1-AS1	[53]	SPRY4-IT1	[6]		
		TUBA4B	[6]	TC0100223	[42]		
		XIST	[54]	TC0101441	[42]		
				TINCR	[51]		
				TTN-AS1	[65]		
				TUBA4B	[6]		
				UCA1	[21]		

**Table 2:** LncRNAs associated with overall survival.

Overall Survival							
LncRNA	Ref.	LncRNA	Ref.	LncRNA	Ref.	LncRNA	Ref.
AB073614	[6,9]	FAM83H-AS1	[15,16]	LINC00504	[70]	MLK7-AS1	[40]
AC104699.1.1	[42]	FEZF1-AS1	[20,71]	LINC00511	[72]	MNX1-AS1	[6]
ADAMTS9-AS2	[19]	FLVCR1-AS1	[41]	LINC00565	[37]	NEAT1	[6]
aHIF	[13]	GAS5	[22,31,46]	LINC01125	[73]	PVT1	[74]
AK021924	[42]	GIHCG	[75]	LINC01127	[76]	RHPN1-AS1	[77]
AK094536	[42]	H19	[42]	LINC01210	[27]	RP11-135L22.1	[48]
ANRIL	[6]	HAGLROS	[29]	LINC01627	[78]	RP11-284N8.3.1	[21]
ASAP1-IT1	[42]	HAS2-AS1	[79]	LNC00908	[80]	RP11-597D13.9	[81]
BC004123	[42]	HMMR-AS1	[49]	LNC01133	[82]	RUNX1-IT1	[42]
BC007937	[42]	HOST2	[83]	lncARSR	[6]	SNHG12	[47]
BC037530	[42]	HOTAIR	[21]	lncBRM	[6]	SNHG14	[84,85,86]
BC062365	[42]	HOTAIRM1	[42]	lnc-HRCT1-1	[42]	SNHG15	[67]
Casc15	[12]	HOTTIP	[52,87]	Lnc-OC1	[6]	SNHG20	[61]
Casc2	[17]	HOXA11-AS	[6]	lnc-SERTAD2-3	[42]	SNHG3	[63,88]
CCAT1	[6]	HOXD-AS1	[6]	LOC100190986	[42]	SNHG5	[62]
CCAT2	[6]	JPX	[25]	LOXL1-AS1	[89]	SPRY4-IT1	[42]
CCEPR	[90]	KCNMA1-AS1	[91]	LUCAT1	[30]	TC0101441	[42,92]
DLX6-AS1	[33]	KCNQ1OT1	[35,93]	MALAT1	[94]	TP73-AS1	[95]
DUXAP10	[42]	LEF1-AS1	[96]	MEG3	[38]	TTN-AS1	[65]
EIBC	[6]	LINC00319	[97]	MIF-AS1	[98]	UCA1	[26]
EPB41L4A-AS2	[23]	LINC00339	[55]	MIR22HG	[6]	XIST	[54,99]
FAM215A	[42]	LINC00472	[21]	miR503HG	[100]	ZFAS1	[101]

**Table 3:** LncRNAs associated with patients’ disease-free and progression-free survival.

Disease-Free Survival		Progression-Free Survival			
LncRNA	Ref.	LncRNA	Ref.	LncRNA	Ref.
DLX6-AS1	[33]	ANRIL	[6]	lnc-HRCT1-1	[42]
GAS5	[6,46]	Casc15	[12]	Lnc-OC1	[6]
H19	[42]	Casc2	[17]	lnc-SERTAD2-3	[42]
HOTAIR	[21]	CCAT1	[6]	MALAT1	[94]
HOTAIRM1	[42]	CCAT2	[6]	MIR22HG	[6]
LINC01210	[27]	EIBC	[6]	MNX1-AS1	[6]
LOC100190986	[42]	FLJ33360	[66]	NEAT1	[6]
MALAT1	[21]	HOXA11-AS	[6]	SNHG15	[67]
RUNX1-IT1	[42]	HOXD-AS1	[6]	SPRY4-IT1	[6]
TC0101441	[92]	lncARSR	[6]	UCA1	[26]
ZFAS1	[101]	lncBRM	[6]	XIST	[42,54]

**Table 4:** LncRNAs associated with resistance to most common chemotherapeutic drugs.

Platinum Salts				Taxanes			
+		–		+		–	
LncRNA	Ref.	LncRNA	Ref.	LncRNA	Ref.	LncRNA	Ref.
ANRIL	[18]	BC200	[21]	CTD-2589M5.4*	[102]	KB-1471A8.2	[103]
CCAT1	[104]	GAS5	[46]	FER1L4	[105]	SNHG5	[62]
CCAT2	[106]	Linc00312	[6]	NEAT1	[105]	XIST	[26]
CTD-2589M5.4*	[102]	LINC00515	[21]	PVT1	[105]		
DNM3OS	[107]	LINC01125	[73]	UCA1	[105,108]		
ENST00000457645	[6]	linc-TNFRSF19-1	[21]				
FER1L4	[105]	MEG3	[42]				
H19	[6,109]	RP11-135L22.1	[48]				
HOTAIR	[105,110]	XIST	[111]				
LINC00152	[112]						
Linc00161	[113]						
LINC00961	[21]						
linc-CARS2-2	[21]						
linc-RECK-3	[21]						
LUCAT1	[21]						
MALAT1	[107]						
NCK1-AS1	[114]						
NEAT1	[105]						
PVT1	[107]						
SNHG15	[67]						
UCA1	[105]						
ZFAS1	[6]						

* Co-expressed with genes associated with multidrug resistance. +, positively associated; −, negatively associated.

**Table 5:** LncRNA–miRNA–mRNA regulatory triplets.

lncRNA	miRNA	mRNA	Ref.
ADAMTS9-AS2	miR-182-5p	FOXF2	[19]
ANRIL	let-7a	HMGA2	[18]
BLACAT1	miR-519d-3p	RPS15A	[142]
Casc15	miR-221	ARID1A	[12]
CCAT1	miR-454	Survivin	[104]
	miR-1290	-	[6]
	miR-130b	STAT3, ZEB1	
	miR-152	ADAM17, WNT1	
CCAT2	miR-424	-	[6]
DANCR	miR-145	VEGF	[133]
DARS-AS1	miR-532-3p	-	[143]
DLEU1	miR-490-3p	-	[32]
DLX6-AS1	miR-613	-	[144]
EPB41L4A-AS2	miR-103a	RUNX1T1	[23]
EWSAT1	miR-330-5p	Pdia3	[6]
FEZF1-AS1	miR-130a-5p	SOX4	[20]
FLJ33360	miR-30b-3p	-	[66]
FLVCRA1-AS1	miR-513	YAP1	[41]
GAS5	miR-196a-5p	HOXA5	[22]
	miR-21	SPRY2	[6]
H19	let-7	HMGA2, c-MYC, IGF2BP	[31]
	miR-324-5p	PKM2	[132]
	miR-370-3p	TGF-B	[26]
HAS2-AS1	miR-466	RUNX2	[79]
HNF1A-AS1	miR-214	SEMA4D, PlexinB1, Tiam1, Rac/1/2/3	[134]
HOST2	let-7b	HMGA2, c-Myc, Dicer, Imp3	[31]
HOTAIR	miR-1	MAPK1	[145]
	miR-200c	-	[146]
	mir-214(-3p)	MAPK1, (PIK3R3)	[145,147]
	miR-217	PIK3R3	[147]
	miR-330-5p	MAPK1	[145]
	miR-373	Rab22a	[6]
HOXD-AS1	miR-133a-3p	-	[6]
	miR-186-5p	PI3KR3	[26]
HOXD-AS1	miR-608	FZD4	[26]
KCNQ1OT1	miR-142-5p	CAPN10	[93]
	miR-212-3p	LCN2	[35]
LINC00152	miR-125b	MCL-1	[6]
Linc00161	miR-194	MAPK1	[113]
LINC00319	miR-423-5p	NACC1	[97]
LINC00339	miR-148a-3p	ROCK1	[55]
LINC00504	miR-1244	-	[70]
LINC01088	miR-24-1-5p	PAK4	[58]
LINC01125	miR-1972	-	[73]
Linc-ROR	miR-145	FLNB	[59]
LNC00908	miR-495-5p	ANXA3	[80]
LNC01133	miR-126	-	[82]
lncARSR	miR-200c	ZEB1, ZEB2	[6]
lncBRM	miR-204	-	[6]
Lnc-OC1	miR-34a	-	[6]
	miR-34c	-	[6]
LOXL1-AS1	miR-18b-5p	VMA21	[89]
LUCAT1	miR-612	HOXA13	[30]
	miR-199a-5p	-	[148]
MAGI1-IT1	miR-200a	ZEB1, ZEB2	[34]
MAGI2-AS3	miR-15b miR-374a miR-374b	HOXA5, MTSS1, PTEN, RECK	[149]
MALAT1	miR-143-3p	CMPK	[94]
	miR-200c	-	[6]
	miR-211	PHF19	[6]
	miR-503-5p	pJak2, pSTAT3	[150]
	miR-506	iASPP	[6]
MCM3AP-AS1	miR-28-5p	-	[36]
MIAT	miR-150-5p	-	[151]
MIF-AS1	miR-315p	PLCB1	[98]
MEG3	miR-214	-	[107]
	miR-219a-5p	EGFR	[38]
	miR-421	PDGFRA, NOTCH1, HES1, RBPJ	[123]
MLK7-AS1	miR-375	YAP1	[40]
NCK1-AS1	miR-137	NCK1	[114]
NEAT1	miR-124	-	[21]
	miR-194	ZEB1	[105]
	miR-382-3p	ROCK1	[6]
	miR-506	RAD51	[105]
NORAD	miR-155-5p	-	[115]
	miR-199a-3p	-	[152]
	miR-608	STAT3	[116]
PCA3	miR-106b	RhoC, Bcl/xL, P70S6K, MMP2	[6]
PTAF	miR-25	SNAI2	[6]
PTAL/AC004988.1	miR-101	FN1	[153]
PTAR	miR-101-3p	ZEB1	[154]
PVT1	miR-133a	-	[6]
	miR-140		[74]
RHPN1-AS1	miR-596-3p	LETM1	[77]
SNHG12	miR-129	SOX4	[47]
SNHG14	miR-125a-5p	DHX33	[86]
	miR-219a-5p	-	[84]
SNHG3	miR-186a-5p miR-590-3p	-	[88]
SNHG5	miR-23a	-	[62]
TDRG1	miR-93	RhoC, P70S6K, Bcl-xL, MMP2	[6]
TINCR	miR-335	FGF2	[51]
TTN-AS1	miR-139-5p	ROCK2	[65]
	miR-15b-5p	FBXW7	[124]
TUG1	miR-29b-3p	MDM2	[155]
UCA1	miR-129	ABCB1	[105]
	miR-485-5p	MMP14	[21]
	miR-654-5p	SIK2	[108]
WDFY3-AS2	miR-18a	RORA	[156]
XIST	miR-150-5p	PDCD4	[99]
	miR-214-3p	PTEN	[105]
ZFAS1	miR-150-5p	Sp1	[6]

**Table 6:** Online databases containing useful information for the study of lncRNAs in ovarian cancer.

Name	Description	Link	Ref.
GEPIA 2	Gene Expression Profiling Interactive Analysis	http://gepia2.cancer-pku.cn/#index	[159]
CCLE	Cancer Cell Line Encyclopedia	https://portals.broadinstitute.org/ccle	[160]
TANRIC	The Atlas of non-coding RNA in Cancer	https://ibl.mdanderson.org/tanric/_design/basic/main.html	[161]
LNCipedia	Database for lncRNA	https://lncipedia.org/	[162]
NONCODE	Database dedicated to ncRNA, especially lncRNA	http://www.noncode.org/	[163]
AnnoLnc	Database	http://annolnc.cbi.pku.edu.cn/index.jsp	[164]
lncRNAdb	Database	http://lncrnadb.org/	[165]
lncRBase	Database	http://bicresources.jcbose.ac.in/zhumur/lncrbase/	[166]
RNAInter	RNA interactome database	http://www.rna-society.org/rnainter	[167]
StarBase-ENCORI	The Encyclopedia of RNA Interactomes	http://starbase.sysu.edu.cn	[168]
miRTarBase	microRNA-target interactions database	http://mirtarbase.mbc.nctu.edu.tw/php/index.php	[169]
ORTI	Open-access Repository of Transcriptional Interaction	http://orti.sydney.edu.au/index.html	[170]
DiseaseMeth	Human disease methylation database	http://bio-bigdata.hrbmu.edu.cn/diseasemeth/	[171]
Lnc2Meth	Relationships between lncRNAs and DNA methylation	http://bio-bigdata.hrbmu.edu.cn/Lnc2Meth/index.jsp	[172]
LncRNA2Target	Database of experiments focused on lncRNA in human and mouse	http://123.59.132.21/lncrna2target/	[173]
CRISPRlnc	Validated CRISPR/Cas9 sgRNAs for lncRNAs from all species	http://www.crisprlnc.org/	[174]
SmProt	Small Proteins (< 100 aa) especially encoded by non-coding RNAs	http://bioinfo.ibp.ac.cn/SmProt/index.htm	[175]
LncTarD	Database for functional lncRNA-target regulation in human diseases	http://biocc.hrbmu.edu.cn/LncTarD/	[176]
LncRNADisease	The LncRNA and Disease Database	http://www.rnanut.net/lncrnadisease/	[177]
LncMAP	LncRNA Modulator Atlas in Pan-cancer	http://bio-bigdata.hrbmu.edu.cn/LncMAP/	[178]
Lnc2Cancer	Experimentally supported associations between lncRNA and human cancer.	http://www.bio-bigdata.com/lnc2cancer/	[179]
CRlncRNA	Cancer-related lncRNA Database	http://crlnc.xtbg.ac.cn	[180]
EMT-Regulome	Database for EMT-related regulatory interactions, motifs and network	http://www.medsysbio.org/EMTRegulome	[181]
RHPCG	Regulation of the Hippo Pathway in Cancer Genome	http://www.medsysbio.org/RHPCG	[182]

**Table 7:** Online tools useful for predicting lncRNA interactions and structures.

Name	Description	Link	Ref.
RPISeq	RNA-Protein Interaction Prediction	http://pridb.gdcb.iastate.edu/RPISeq/	[183]
RPI-Pred	RNA-protein interaction prediction server	http://ctsb.is.wfubmc.edu/projects/rpi-pred/	[184]
lncPro	Prediction of lncRNA-protein interactions	http://bioinfo.bjmu.edu.cn/lncpro/	[185]
catRAPID	Algorithm to estimate the binding propensity of protein-RNA pairs	http://s.tartaglialab.com/page/catrapid_group	[186]
mirDIP	microRNA Data Integration Portal	http://ophid.utoronto.ca/mirDIP/	[187]
LncRRISearch	lncRNA-RNA interaction prediction	http://rtools.cbrc.jp/LncRRIsearch/	[188]
LongTarget & LongMan	Predict a lncRNA’s DNA binding motifs and binding sites, locally or at genome-wide scale	http://lncrna.smu.edu.cn/	[189]
RNAfold web server	Predicts RNA secondary structures	http://rna.tbi.univie.ac.at/cgi-bin/RNAWebSuite/RNAfold.cgi	[190]

## Supplementary Materials

The following are available online at https://www.mdpi.com/2072-6694/12/4/1020/s1. Table S1: Overall experimental analyses of lncRNAs in OC (date 24/03/2020).

## References

[1] Bray F., Ferlay J., Soerjomataram I., Siegel R.L., Torre L.A., Jemal A. (2018). Global cancer statistics 2018: GLOBOCAN estimates of incidence and mortality worldwide for 36 cancers in 185 countries. CA Cancer J. Clin..

[2] Brett M.R., Jennifer B.P., Thomas A.S., Brett M.R., Jennifer B.P., Thomas A.S. (2017). Epidemiology of ovarian cancer: A review. Cancer Biol. Med..

[3] Ueland F. (2017). A Perspective on Ovarian Cancer Biomarkers: Past, Present and Yet-To-Come. Diagnostics.

[4] Cortez A.J., Tudrej P., Kujawa K.A., Lisowska K.M. (2018). Advances in ovarian cancer therapy. Cancer Chemother. Pharmacol..

[5] Wang K.C., Chang H.Y. (2011). Molecular Mechanisms of Long Noncoding RNAs. Mol. Cell.

[6] Wang J., Lu A., Chen L. (2019). LncRNAs in ovarian cancer. Clin. Chim. Acta.

[7] Iyer M.K., Niknafs Y.S., Malik R., Singhal U., Sahu A., Hosono Y., Barrette T.R., Prensner J.R., Evans J.R., Zhao S. (2015). The landscape of long noncoding RNAs in the human transcriptome. Nat. Genet..

[8] Hu X., Bao J., Wang Z., Zhang Z., Gu P., Tao F., Cui D., Jiang W. (2016). The plasma lncRNA acting as fingerprint in non-small-cell lung cancer. Tumor Biol..

[9] Zeng S., Liu S., Feng J., Gao J., Xue F. (2019). Upregulation of lncRNA AB073614 functions as a predictor of epithelial ovarian cancer prognosis and promotes tumor growth in vitro and in vivo. Cancer Biomark..

[10] Yang M., Zhai Z., Zhang Y., Wang Y. (2019). Clinical significance and oncogene function of long noncoding RNA HAGLROS overexpression in ovarian cancer. Arch. Gynecol. Obstet..

[11] Lin X., Tang X., Zheng T., Qiu J., Hua K. (2019). Long non-coding RNA NONHSAT076754 promotes invasion and metastasis in epithelial ovarian cancer. J. Cancer.

[12] Wu D.-D., Chen X., Sun K.-X., Wang L.-L., Chen S., Zhao Y. (2017). Role of the lncRNA ABHD11-AS1 in the tumorigenesis and progression of epithelial ovarian cancer through targeted regulation of RhoC. Mol. Cancer.

[13] Yu H., Xu Y., Zhang D., Liu G. (2018). Long noncoding RNA LUCAT1 promotes malignancy of ovarian cancer through regulation of miR-612/HOXA13 pathway. Biochem. Biophys. Res. Commun..

[14] Long X., Song K., Hu H., Tian Q., Wang W., Dong Q., Yin X., Di W. (2019). Long non-coding RNA GAS5 inhibits DDP-resistance and tumor progression of epithelial ovarian cancer via GAS5-E2F4-PARP1-MAPK axis. J. Exp. Clin. Cancer Res..

[15] Tang X., Liu S., Liu Y., Lin X., Zheng T., Liu X., Qiu J., Hua K. (2019). Circulating serum exosomal aHIF is a novel prognostic predictor for epithelial ovarian cancer. Onco. Targets. Ther..

[16] Gao H., Li X., Zhan G., Zhu Y., Yu J., Wang J., Li L., Wu W., Liu N., Guo X. (2019). Long noncoding RNA MAGI1-IT1 promoted invasion and metastasis of epithelial ovarian cancer via the miR-200a/ZEB axis. Cell Cycle.

[17] Pei C.L., Fei K.L., Yuan X.Y., Gong X.J. (2019). LncRNA DANCR aggravates the progression of ovarian cancer by downregulating UPF1. Eur. Rev. Med. Pharmacol. Sci..

[18] Worku T., Bhattarai D., Ayers D., Wang K., Wang C., Rehman Z.U., Talpur H.S., Yang L. (2017). Long Non-Coding RNAs: The New Horizon of Gene Regulation in Ovarian Cancer. Cell. Physiol. Biochem..

[19] Sun D., Fan X.H. (2019). LncRNA SNHG12 accelerates the progression of ovarian cancer via absorbing miRNA-129 to upregulate SOX4. Eur. Rev. Med. Pharmacol. Sci..

[20] Lu X., Wang F., Fu M., Li Y., Wang L. (2019). Long non-coding RNA KCNQ1OT1 accelerates the progression of ovarian cancer via microRNA-212-3/ LCN2 axis. Oncol. Res. Featur. Preclin. Clin. Cancer Ther..

[21] Wang L.-L., Sun K.-X., Wu D.-D., Xiu Y.-L., Chen X., Chen S., Zong Z.-H., Sang X.-B., Liu Y., Zhao Y. (2017). DLEU1 contributes to ovarian carcinoma tumourigenesis and development by interacting with miR-490-3p and altering CDK1 expression. J. Cell. Mol. Med..

[22] Lou Y., Jiang H., Cui Z., Wang X., Wang L., Han Y. (2018). Gene microarray analysis of lncRNA and mRNA expression profiles in patients with high-grade ovarian serous cancer. Int. J. Mol. Med..

[23] Shi Y., Gao S., Zheng Y., Yao M., Ruan F. (2019). LncRNA CASC15 Functions As An Unfavorable Predictor Of Ovarian Cancer Prognosis And Inhibits Tumor Progression Through Regulation Of miR-221/ARID1A Axis. Onco. Targets. Ther..

[24] Gong Y.B., Zou Y.F. (2019). Clinical significance of lncRNA FAM83H-AS1 in ovarian cancer. Eur. Rev. Med. Pharmacol. Sci..

[25] Wang A., Jin C., Li H., Qin Q., Li L. (2018). LncRNA ADAMTS9-AS2 regulates ovarian cancer progression by targeting miR-182-5p/FOXF2 signaling pathway. Int. J. Biol. Macromol..

[26] Dou Q., Xu Y., Zhu Y., Hu Y., Yan Y., Yan H. (2019). LncRNA FAM83H-AS1 contributes to the radioresistance, proliferation, and metastasis in ovarian cancer through stabilizing HuR protein. Eur. J. Pharmacol..

[27] Wang D., Dai J., Hou S., Qian Y. (2019). LncRNA SNHG20 predicts a poor prognosis and promotes cell progression in epithelial ovarian cancer. Biosci. Rep..

[28] Sun T., Yang P., Gao Y. (2020). Long non-coding RNA EPB41L4A-AS2 suppresses progression of ovarian cancer by sequestering microRNA-103a to upregulate transcription factor RUNX1T1. Exp. Physiol..

[29] Xue Z., Zhu X., Teng Y. (2018). Long non-coding RNA CASC2 inhibits progression and predicts favorable prognosis in epithelial ovarian cancer. Mol. Med. Rep..

[30] Lin H., Shen L., Lin Q., Dong C., Maswela B., Illahi G.S., Wu X. (2020). SNHG5 enhances Paclitaxel sensitivity of ovarian cancer cells through sponging miR-23a. Biomed. Pharmacother..

[31] Miao J.-T., Gao J.-H., Chen Y.-Q., Chen H., Meng H.-Y., Lou G. (2019). LncRNA ANRIL affects the sensitivity of ovarian cancer to cisplatin via regulation of let-7a/HMGA2 axis. Biosci. Rep..

[32] Zhao J., Liu H.R. (2019). Down-regulation of long noncoding RNA DLX6-AS1 defines good prognosis and inhibits proliferation and metastasis in human epithelial ovarian cancer cells via Notch signaling pathway. Eur. Rev. Med. Pharmacol. Sci..

[33] Zou S.H., Du X., Sun F.D., Wang P.C., Li M. (2018). Cisplatin suppresses tumor proliferation by inhibiting autophagy in ovarian cancer via long non-coding RNA RP11-135L22.1. Eur. Rev. Med. Pharmacol. Sci..

[34] Hong L., Chen W., Wu D., Wang Y. (2018). Upregulation of SNHG3 expression associated with poor prognosis and enhances malignant progression of ovarian cancer. Cancer Biomark..

[35] Sun Z., Gao S., Xuan L., Liu X. (2020). Long non-coding RNA FEZF1-AS1 induced progression of ovarian cancer via regulating miR-130a-5p/SOX4 axis. J. Cell. Mol. Med..

[36] Zuo K., Zhao Y., Zheng Y., Chen D., Liu X., Du S., Liu Q. (2019). Long non-coding RNA XIST promotes malignant behavior of epithelial ovarian cancer. Onco. Targets. Ther..

[37] Tripathi M.K., Doxtater K., Keramatnia F., Zacheaus C., Yallapu M.M., Jaggi M., Chauhan S.C. (2018). Role of lncRNAs in ovarian cancer: Defining new biomarkers for therapeutic purposes. Drug Discov. Today.

[38] Yan H., Li H., Silva M.A., Guan Y., Yang L., Zhu L., Zhang Z., Li G., Ren C. (2019). LncRNA FLVCR1-AS1 mediates miR-513/YAP1 signaling to promote cell progression, migration, invasion and EMT process in ovarian cancer. J. Exp. Clin. Cancer Res..

[39] Zhao H., Yu H., Zheng J., Ning N., Tang F., Yang Y., Wang Y. (2018). Lowly-expressed lncRNA GAS5 facilitates progression of ovarian cancer through targeting miR-196-5p and thereby regulating HOXA5. Gynecol. Oncol..

[40] Pan L., Meng Q., Li H., Liang K., Li B. (2019). LINC00339 promotes cell proliferation, migration, and invasion of ovarian cancer cells via miR-148a-3p/ROCK1 axes. Biomed. Pharmacother..

[41] Chu Z.P., Dai J., Jia L.G., Li J., Zhang Y., Zhang Z.Y., Yan P. (2018). Increased expression of long noncoding RNA HMMR-AS1 in epithelial ovarian cancer: An independent prognostic factor. Eur. Rev. Med. Pharmacol. Sci..

[42] Gong M., Luo C., Meng H., Li S., Nie S., Jiang Y., Wan Y., Li H., Cheng W. (2019). Upregulated LINC00565 Accelerates Ovarian Cancer Progression By Targeting GAS6. Onco. Targets. Ther..

[43] Liu D., Li H. (2019). Long non-coding RNA GEHT1 promoted the proliferation of ovarian cancer cells via modulating the protein stability of HIF1α. Biosci. Rep..

[44] Ni M.-W., Zhou J., Zhang Y.-L., Zhou G.-M., Zhang S.-J., Feng J.-G., Xu Q., Zhou Y., Mou H.-Z., Zheng Z.-G. (2019). Downregulation of LINC00515 in high-grade serous ovarian cancer and its relationship with platinum resistance. Biomark. Med..

[45] Li J., Feng L., Tian C., Tang Y.L., Tang Y., Hu F.Q. (2018). Long noncoding RNA-JPX predicts the poor prognosis of ovarian cancer patients and promotes tumor cell proliferation, invasion and migration by the PI3K/Akt/mTOR signaling pathway. Eur. Rev. Med. Pharmacol. Sci..

[46] Zhu Y., Shi L., Zhou C., Wang Z., Yu T., Zhou J., Yang Y. (2019). Long non-coding RNA MCM3AP-AS1 inhibits cell viability and promotes apoptosis in ovarian cancer cells by targeting miR-28-5p. Int. J. Clin. Exp. Med..

[47] Guo Q., Wang L., Zhu L., Lu X., Song Y., Sun J., Wu Z., Shi J., Wang Z., Zhou X. (2019). The clinical significance and biological function of lncRNA SOCAR in serous ovarian carcinoma. Gene.

[48] Oncul S., Amero P., Rodriguez-Aguayo C., Calin G.A., Sood A.K., Lopez-Berestein G. (2019). Long non-coding RNAs in ovarian cancer: Expression profile and functional spectrum. Rna Biol..

[49] He S., Zhao Y., Wang X., Deng Y., Wan Z., Yao S., Shen H. (2018). Up-regulation of long non-coding RNA SNHG20 promotes ovarian cancer progression via Wnt/β-catenin signaling. Biosci. Rep..

[50] Liu X., Li Y., Wen J., Qi T., Wang Y. (2020). Long non-coding RNA TTN-AS1 promotes tumorigenesis of ovarian cancer through modulating the miR-139-5p/ROCK2 axis. Biomed. Pharmacother..

[51] Zhang C., Liu J., Zhang Y., Luo C., Zhu T., Zhang R., Yao R. (2019). LINC01210 accelerates proliferation, invasion and migration in ovarian cancer through epigenetically downregulating KLF4. Biomed. Pharmacother..

[52] Li R., Wang Y., Xu Y., He X., Li Y. (2019). Silencing the long noncoding RNA, TINCR, a molecular sponge of miR-335, inhibits the malignant phenotype of epithelial ovarian cancer via FGF2 suppression. Int. J. Oncol..

[53] Liu Y., Wang Y., Yao D., Cui D. (2018). LncSOX4 serves an oncogenic role in the tumorigenesis of epithelial ovarian cancer by promoting cell proliferation and inhibiting apoptosis. Mol. Med. Rep..

[54] Ding C., Wei R., Rodríguez R.A., Requena M. (2019). LncRNA PCAT-1 plays an oncogenic role in epithelial ovarian cancer by modulating cyclinD1/CDK4 expression. Int. J. Clin. Exp. Pathol..

[55] Wang L., Yu M., Zhao S. (2019). lncRNA MEG3 modified epithelial-mesenchymal transition of ovarian cancer cells by sponging miR-219a-5p and regulating EGFR. J. Cell. Biochem..

[56] Zou T., Wang P.L., Gao Y., Liang W.T. (2019). Long noncoding RNA HOTTIP is a significant indicator of ovarian cancer prognosis and enhances cell proliferation and invasion. Cancer Biomark..

[57] Wu W., Gao H., Li X., Zhu Y., Peng S., Yu J., Zhan G., Wang J., Liu N., Guo X. (2019). LncRNA TPT1-AS1 promotes tumorigenesis and metastasis in epithelial ovarian cancer by inducing TPT1 expression. Cancer Sci..

[58] Wang L., Ye T.Y., Wu H., Chen S.Y., Weng J.R., Xi X.W. (2019). LINC00702 accelerates the progression of ovarian cancer through interacting with EZH2 to inhibit the transcription of KLF2. Eur. Rev. Med. Pharmacol. Sci..

[59] Yan H., Li H., Li P., Li X., Lin J., Zhu L., Silva M.A., Wang X., Wang P., Zhang Z. (2018). Long noncoding RNA MLK7-AS1 promotes ovarian cancer cells progression by modulating miR-375/YAP1 axis. J. Exp. Clin. Cancer Res..

[60] Zhan L., Li J., Wei B. (2018). Long non-coding RNAs in ovarian cancer. J. Exp. Clin. Cancer Res..

[61] Chen X., Wu W., Cao X., Zhao X., Li W., Deng C., Huang Z. (2019). lncRNA mortal obligate RNA transcript was downregulated in ovarian carcinoma and inhibits cancer cell proliferation by downregulating miRNA-21. J. Cell. Biochem..

[62] Kong F.R., Lv Y.H., Yao H.M., Zhang H.Y., Zhou Y., Liu S.E. (2019). LncRNA PCAT6 promotes occurrence and development of ovarian cancer by inhibiting PTEN. Eur. Rev. Med. Pharmacol. Sci..

[63] Lou Y., Jiang H., Cui Z., Wang L., Wang X., Tian T. (2017). Linc-ROR induces epithelial-to-mesenchymal transition in ovarian cancer by increasing Wnt/β-catenin signaling. Oncotarget.

[64] Zhang W., Fei J., Yu S., Shen J., Zhu X., Sadhukhan A., Lu W., Zhou J. (2018). LINC01088 inhibits tumorigenesis of ovarian epithelial cells by targeting miR-24-1-5p. Sci. Rep..

[65] Zhang S., Li J., Wu L., Pei M. (2019). Interaction between LncRNA-ROR and miR-145 contributes to epithelial-mesenchymal transition of ovarian cancer cells. Gen. Physiol. Biophys..

[66] Yang M., Zhai Z., Guo S., Li X., Zhu Y., Wang Y. (2019). Long non-coding RNA FLJ33360 participates in ovarian cancer progression by sponging miR-30b-3p. Onco. Targets. Ther..

[67] Qu C., Dai C., Guo Y., Qin R., Liu J. (2018). Long noncoding RNA SNHG15 serves as an oncogene and predicts poor prognosis in epithelial ovarian cancer. Onco. Targets. Ther..

[68] Prat J. (2014). Staging classification for cancer of the ovary, fallopian tube, and peritoneum. Int. J. Gynecol. Obstet..

[69] Chen J., Peng X., Dai Y. (2019). The long non-coding RNA (lncRNA) AGAP2-AS1 is upregulated in ovarian carcinoma and negatively regulates lncRNA MEG3. Med. Sci. Monit..

[70] Jiang J.N., Hong Q.Y., Gao H.J., Lai B.L., Lan J.F., Yang Q. (2019). Lnc00908 promotes the development of ovarian cancer by regulating microRNA-49. Eur. Rev. Med. Pharmacol. Sci..

[71] Zhao X., Cheng Z., Wang J. (2018). Long noncoding RNA FEZF1-AS1 promotes proliferation and inhibits apoptosis in ovarian cancer by activation of JAK-STAT3 pathway. Med. Sci. Monit..

[72] Gu X., Xu P., Xu S. (2018). LncRNA RP11-597D13.9 expression and clinical significance in serous Ovarian Cancer based on TCGA database. Cancer Cell Res..

[73] Hou B., Hou X., Ni H. (2018). Long non-coding RNA LNC01133 promotes the tumorigenesis of ovarian cancer by sponging miR-126. Int. J. Clin. Exp. Pathol..

[74] Ma S.Y., Wei P., Qu F. (2019). KCNMA1-AS1 attenuates apoptosis of epithelial ovarian cancer cells and serves as a risk factor for poor prognosis of epithelial ovarian cancer. Eur. Rev. Med. Pharmacol. Sci..

[75] Wang J., Tian Y., Zheng H., Ding Y., Wang X. (2019). An integrated analysis reveals the oncogenic function of lncRNA LINC00511 in human ovarian cancer. Cancer Med..

[76] Xu Y., Jiang T., Wang C., Wang F. (2019). Sinomenine hydrochloride exerts antitumor outcome in ovarian cancer cells by inhibition of long non-coding RNA HOST2 expression. Artif. Cellsnanomedicinebiotechnol..

[77] Qiu J., Lin X., Tang X., Zheng T., Zhang X., Hua K. (2020). Long noncoding RNA TC0101441 induces epithelial–mesenchymal transition in epithelial ovarian cancer metastasis by downregulating KiSS1. Int. J. Cancer.

[78] Li L., Zhang R., Li S.J. (2019). Long noncoding RNA SNHG14 promotes ovarian cancer cell proliferation and metastasis via sponging miR-219a-5p. Eur. Rev. Med. Pharmacol. Sci..

[79] Guo J., Pan H. (2019). Long noncoding RNA LINC01125 enhances cisplatin sensitivity of ovarian cancer via miR-1972. Med. Sci. Monit..

[80] Zhao J., Wang C., Liu Y., Zhang G. (2019). Long noncoding RNA SNHG14 enhances migration and invasion of ovarian cancer by upregulating DGCR8. Eur. Rev. Med. Pharmacol. Sci..

[81] Liu H., Chen R., Kang F., Lai H., Wang Y. (2020). KCNQ1OT1 promotes ovarian cancer progression via modulating MIR-142-5p / CAPN10 axis. Mol. Genet. Genom. Med..

[82] Zhao Y.L., Huang Y.M. (2019). LncSNHG14 promotes ovarian cancer by targeting microRNA-125a-5p. Eur. Rev. Med. Pharmacol. Sci..

[83] Ding Y., Fang Q., Li Y., Wang Y. (2019). Amplification of lncRNA PVT1 promotes ovarian cancer proliferation by binding to miR-140. Mamm. Genome.

[84] Lin Q., Guan W., Ren W., Zhang L., Zhang J., Xu G. (2018). MALAT1 affects ovarian cancer cell behavior and patient survival. Oncol. Rep..

[85] Li Y., Jiao Y., Hao J., Xing H., Li C. (2019). Long noncoding RNA TP73-AS1 accelerates the epithelial ovarian cancer via epigenetically repressing p21. Am. J. Transl. Res..

[86] Zhang Y., Ruan F. (2020). LncRNA LEF1-AS1 Promotes Ovarian Cancer Development Through Interacting with miR-1285-3p. Cancer Manag. Res..

[87] Yao N., Yu L., Zhu B., Gan H.Y., Guo B.Q. (2018). LncRNA GIHCG promotes development of ovarian cancer by regulating microRNA-429. Eur. Rev. Med. Pharmacol. Sci..

[88] Du W., Feng Z., Sun Q. (2018). LncRNA LINC00319 accelerates ovarian cancer progression through miR-423-5p/NACC1 pathway. Biochem. Biophys. Res. Commun..

[89] Shang A., Wang W., Gu C., Chen C., Zeng B., Yang Y., Ji P., Sun J., Wu J., Lu W. (2019). Long non-coding RNA HOTTIP enhances IL-6 expression to potentiate immune escape of ovarian cancer cells by upregulating the expression of PD-L1 in neutrophils. J. Exp. Clin. Cancer Res..

[90] Liu Y., He X., Chen Y., Cao D. (2020). Long non-coding RNA LINC00504 regulates the Warburg effect in ovarian cancer through inhibition of miR-1244. Mol. Cell. Biochem..

[91] Jing L., Gong M., Lu X., Jiang Y., Li H., Cheng W. (2019). LINC01127 promotes the development of ovarian tumors by regulating the cell cycle. Am. J. Transl. Res..

[92] Fan Y., Wang L., Han X.-C., Ma H.-Y., Zhang N., Zhe L. (2020). LncRNA MIF-AS1 aggravates the progression of ovarian cancer by sponging miRNA-31-5p. Eur. Rev. Med. Pharmacol. Sci..

[93] Wang J., Ding W., Xu Y., Tao E., Mo M., Xu W., Cai X., Chen X., Yuan J., Wu X. (2020). Long non-coding RNA RHPN1-AS1 promotes tumorigenesis and metastasis of ovarian cancer by acting as a ceRNA against miR-596 and upregulating LETM1. Aging (Albany NY).

[94] Li N., Zhan X., Zhan X. (2018). The lncRNA SNHG3 regulates energy metabolism of ovarian cancer by an analysis of mitochondrial proteomes. Gynecol. Oncol..

[95] Wang S., Li G. (2020). LncRNA XIST inhibits ovarian cancer cell growth and metastasis via regulating miR-150-5p/PDCD4 signaling pathway. Naunyn. Schmiedebergs. Arch. Pharmacol..

[96] Shen X., Zhu W. (2019). Long non-coding RNA LINC01627 is a prognostic risk factor for epithelial ovarian cancer. Oncol. Lett..

[97] Tong L., Wang Y., Ao Y., Sun X. (2019). CREB1 induced lncRNA HAS2-AS1 promotes epithelial ovarian cancer proliferation and invasion via the miR-466/RUNX2 axis. Biomed. Pharmacother..

[98] Xue F., Xu Y.H., Shen C.C., Qin Z.L., Zhou H. (2020). Bin Non-coding RNA LOXL1-AS1 exhibits oncogenic activity in ovarian cancer via regulation of miR-18b-5p/VMA21 axis. Biomed. Pharmacother..

[99] Zhu D., Huang X., Liang F., Zhao L. (2020). LncRNA miR503HG interacts with miR-31-5p through multiple ways to regulate cancer cell invasion and migration in ovarian cancer. J. Ovarian Res..

[100] Chen Z., Zhu Y., Fan X., Liu Y., Feng Q. (2020). Upregulation of long non-coding RNA CCEPR is associated with poor prognosis and contributes to the progression of ovarian cancer through regulating the Wnt/β-catenin signaling pathway. Mol. Med. Rep..

[101] Han S., Li D., Xiao M. (2019). LncRNA ZFAS1 serves as a prognostic biomarker to predict the survival of patients with ovarian cancer. Exp. Ther. Med..

[102] Wang D.-Y., Li N., Cui Y.-L. (2020). Long non-coding RNA CCAT1 sponges miR-454 to promote chemoresistance of ovarian cancer cells to cisplatin by regulation of surviving. Cancer Res. Treat..

[103] Chang H., Li B., Zhang X., Meng X. (2020). NCK1-AS1 promotes NCK1 expression to facilitate tumorigenesis and chemo-resistance in ovarian cancer. Biochem. Biophys. Res. Commun..

[104] Xu J., Wu J., Fu C., Teng F., Liu S., Dai C., Shen R., Jia X. (2018). Multidrug resistant lncRNA profile in chemotherapeutic sensitive and resistant ovarian cancer cells. J. Cell. Physiol..

[105] Li W., Han X., Pan Y., Lan Y., Ning J., Zhao Y., Zhang L. (2019). Long non-coding RNA CCAT2 regulates proliferation, drug sensitivity and metastasis of ovarian cancer. Arch. Med. Sci..

[106] Zhang M., Liu S., Fu C., Wang X., Zhang M., Liu G., Dai C., Gong Z., Xu H., Fu Z. (2019). LncRNA KB-1471A8.2 overexpression suppresses cell proliferation and migration and antagonizes the paclitaxel resistance of ovarian cancer cells. Cancer Biother. Radiopharm..

[107] Wambecke A., Ahmad M., Lambert B., Joly F., Poulain L., Denoyelle C., Meryet-Figuiere M. (2020). The influence of long non-coding RNAs on the response to chemotherapy in ovarian cancer. Gynecol. Oncol..

[108] Xu M., Zhou K., Wu Y., Wang L., Lu S. (2019). Linc00161 regulated the drug resistance of ovarian cancer by sponging microRNA-128 and modulating MAPK1. Mol. Carcinog..

[109] Qu J., Kamal M.A., Yuan C. (2019). The Regulatory Roles of Long Non-Coding RNA in the Chemoresistance Process of Ovarian cancer. Curr. Pharm. Des..

[110] Li Z.Y., Wang X.L., Dang Y., Zhu X.Z., Zhang Y.H., Cai B.X., Zheng L. (2020). Long non-coding RNA UCA1 promotes the progression of paclitaxel resistance in ovarian cancer by regulating the miR-654-5p/SIK2 axis. Eur. Rev. Med. Pharmacol. Sci..

[111] Zou H., Li H. (2019). Knockdown of long non-coding RNA LINC00152 increases cisplatin sensitivity in ovarian cancer cells. Exp. Ther. Med..

[112] Wu Y., Zhou Y., He J., Sun H., Jin Z. (2019). Long non-coding RNA H19 mediates ovarian cancer cell cisplatin-resistance and migration during EMT. Int. J. Clin. Exp. Pathol..

[113] Özeş A.R., Wang Y., Zong X., Fang F., Pilrose J., Nephew K.P. (2017). Therapeutic targeting using tumor specific peptides inhibits long non-coding RNA HOTAIR activity in ovarian and breast cancer. Sci. Rep..

[114] Nikpayam E., Tasharrofi B., Sarrafzadeh S., Ghafouri-Fard S. (2017). The role of long non-coding RNAs in ovarian cancer. Iran. Biomed. J..

[115] Tong L., Ao Y., Zhang H., Wang K., Wang Y., Ma Q. (2019). Long noncoding RNA NORAD is upregulated in epithelial ovarian cancer and its downregulation suppressed cancer cell functions by competing with miR-155-5p. Cancer Med..

[116] Yang X., Yan Y., Chen Y., Li J., Yang J. (2019). Involvement of NORAD/miR-608/STAT3 axis in carcinostasis effects of physcion 8-O-β-glucopyranoside on ovarian cancer cells. Artif. Cells Nanomed. Biotechnol..

[117] Park S.-A., Kim L.K., Kim H.J. (2019). Long non-coding RNA E2F4as promotes tumor progression and predicts patient prognosis in human ovarian cancer. Ann. Oncol..

[118] ClinicalTrials.gov. https://clinicaltrials.gov/ct2/show/NCT03738319.

[119] ClinicalTrials.gov. https://clinicaltrials.gov/ct2/show/NCT03742856.

[120] Frankish A., Diekhans M., Ferreira A.-M., Johnson R., Jungreis I., Loveland J., Mudge J.M., Sisu C., Wright J., Armstrong J. (2019). GENCODE reference annotation for the human and mouse genomes. Nucleic Acids Res..

[121] DiStefano J.K. (2018). The Emerging Role of Long Noncoding RNAs in Human Disease. Disease Gene Identification. Methods in Molecular Biology.

[122] Wang J., Xu W., He Y., Xia Q., Liu S. (2018). LncRNA MEG3 impacts proliferation, invasion, and migration of ovarian cancer cells through regulating PTEN. Inflamm. Res..

[123] Ye W., Ni Z., Yicheng S., Pan H., Huang Y., Xiong Y., Liu T. (2019). Anisomycin inhibits angiogenesis in ovarian cancer by attenuating the molecular sponge effect of the lncRNA-Meg3/miR-421/PDGFRA axis. Int. J. Oncol..

[124] Miao S., Wang J., Xuan L., Liu X. (2020). LncRNA TTN-AS1 acts as sponge for miR-15b-5p to regulate FBXW7 expression in ovarian cancer. BioFactors.

[125] Hanahan D., Weinberg R.A. (2011). Hallmarks of Cancer: The Next Generation. Cell.

[126] Wu K., Li L., Li L., Wang D. (2020). Long non-coding RNA HAL suppresses the migration and invasion of serous ovarian cancer by inhibiting EMT signaling pathway. Biosci. Rep..

[127] Yuan D., Zhang X., Zhao Y., Qian H., Wang H., He C., Liu X., Guo T., Lin M., Yu H. (2019). Role of lncRNA-ATB in ovarian cancer and its mechanisms of action. Exp. Ther. Med..

[128] Li J., Yang C., Li Y., Chen A., Li L., You Z. (2018). LncRNA GAS5 suppresses ovarian cancer by inducing inflammasome formation. Biosci. Rep..

[129] Yun C., Lee S. (2018). The Roles of Autophagy in Cancer. Int. J. Mol. Sci..

[130] Zeisberg M., Neilson E.G. (2009). Biomarkers for epithelial-mesenchymal transitions. J. Clin. Invest..

[131] Lu Y., Hu Z., Mangala L.S., Stine Z.E., Hu X., Jiang D., Xiang Y., Zhang Y., Pradeep S., Rodriguez-Aguayo C. (2018). MYC Targeted Long Noncoding RNA DANCR Promotes Cancer in Part by Reducing p21 Levels. Cancer Res..

[132] Zheng X., Zhou Y., Chen W., Chen L., Lu J., He F., Li X., Zhao L. (2018). Ginsenoside 20(S)-Rg3 Prevents PKM2-Targeting miR-324-5p from H19 Sponging to Antagonize the Warburg Effect in Ovarian Cancer Cells. Cell. Physiol. Biochem..

[133] Lin X., Yang F., Qi X., Li Q., Wang D., Yi T., Yin R., Zhao X., Zhong X., Bian C. (2019). LncRNA DANCR promotes tumor growth and angiogenesis in ovarian cancer through direct targeting of miR-145. Mol. Carcinog..

[134] Liu Y., Lin J., Pan J., Qing Q., Li D., Liao J., Sun C., Zhou H. (2019). LncRNA HNF1A-AS1 promotes ovarian cancer growth by countering miR-214-mediated suppression of the sema 4D/plexin B1 pathway. Lancet.

[135] Charbonneau B., Block M.S., Bamlet W.R., Vierkant R.A., Kalli K.R., Fogarty Z., Rider D.N., Sellers T.A., Tworoger S.S., Poole E. (2014). Risk of Ovarian Cancer and the NF- B Pathway: Genetic Association with IL1A and TNFSF10. Cancer Res..

[136] Zeng X., Jiang X.-Y., Yong J.-H., Xie H., Yuan J., Zeng D., Dou Y.-Y., Xiao S.-S. (2019). lncRNA ABHD11-AS1, regulated by the EGFR pathway, contributes to the ovarian cancer tumorigenesis by epigenetically suppressing TIMP2. Cancer Med..

[137] Wang H., Su H., Tan Y. (2020). UNC5B-AS1 promoted ovarian cancer progression by regulating the H3K27me on NDRG2 via EZH2. Cell Biol. Int..

[138] Guo L., Wang S. (2019). Downregulated Long Noncoding RNA GAS5 Fails to Function as Decoy of CEBPB, Resulting in Increased GDF15 Expression and Rapid Ovarian Cancer Cell Proliferation. Cancer Biother. Radiopharm..

[139] Dai L., Niu J., Feng Y. (2020). Knockdown of long non-coding RNA LINC00176 suppresses ovarian cancer progression by BCL3-mediated down-regulation of ceruloplasmin. J. Cell. Mol. Med..

[140] Drak Alsibai K., Vacher S., Meseure D., Nicolas A., Lae M., Schnitzler A., Chemlali W., Cros J., Longchampt E., Cacheux W. (2019). High Positive Correlations between ANRIL and p16-CDKN2A/p15-CDKN2B/p14-ARF Gene Cluster Overexpression in Multi-Tumor Types Suggest Deregulated Activation of an ANRIL–ARF Bidirectional Promoter. Non-Coding Rna.

[141] Zhang S., Leng T., Zhang Q., Zhao Q., Nie X., Yang L. (2018). Sanguinarine inhibits epithelial ovarian cancer development via regulating long non-coding RNA CASC2-EIF4A3 axis and/or inhibiting NF-κB signaling or PI3K/AKT/mTOR pathway. Biomed. Pharmacother..

[142] Yang H., Qi Y., Wang X., Gu J., Shi T. (2020). Down-regulation of lncRNA BLACAT1 inhibits ovarian cancer progression by suppressing the Wnt/β-catenin signaling pathway via regulating miR-519d-3p. Mol. Cell. Biochem..

[143] Huang K., Fan W.S., Fu X.Y., Li Y.L., Meng Y.G. (2019). Long noncoding RNA DARS-AS1 acts as an oncogene by targeting miR-532-3p in ovarian cancer. Eur. Rev. Med. Pharmacol. Sci..

[144] You Q., Shi H.-Y., Gong C.-F., Tian X.-Y., Li S. (2019). Long non-coding RNA DLX6-AS1 acts as an oncogene by targeting miR-613 in ovarian cancer. Eur. Rev. Med. Pharmacol. Sci..

[145] Yiwei T., Hua H., Hui G., Mao M., Xiang L. (2015). HOTAIR Interacting with MAPK1 Regulates Ovarian Cancer skov3 Cell Proliferation, Migration, and Invasion. Med. Sci. Monit..

[146] Yang C., Li H., Zhang T., Chu Y., Chen D., Zuo J. (2020). miR-200c overexpression inhibits the invasion and tumorigenicity of epithelial ovarian cancer cells by suppressing lncRNA HOTAIR in mice. J. Cell. Biochem..

[147] Dong L., Hui L. (2016). HOTAIR promotes proliferation, migration, and invasion of ovarian cancer SKOV3 cells through regulating PIK3R3. Med. Sci. Monit..

[148] Liu H., Liu G., Pang W., Zhang H., Zeng Z., Wang H. (2020). LncRNA LUCAT1 promotes proliferation of ovarian cancer cells by regulating miR-199a-5p expression. Eur. Rev. Med. Pharmacol. Sci..

[149] Gokulnath P., De Cristofaro T., Manipur I., Di Palma T., Soriano A.A., Guarracino M.R., Zannini M. (2019). Long Non-Coding RNA MAGI2-AS3 is a New Player with a Tumor Suppressive Role in High Grade Serous Ovarian Carcinoma. Cancers.

[150] Sun Q., Li Q., Xie F. (2019). LncRNA-MALAT1 regulates proliferation and apoptosis of ovarian cancer cells by targeting miR-503-5p. Onco. Targets. Ther..

[151] Zhou S., Xu A., Song T., Gao F., Sun H., Kong X. (2020). lncRNA MIAT Regulates Cell Growth, Migration, and Invasion Through Sponging miR-150-5p in Ovarian Cancer. Cancer Biother. Radiopharm..

[152] Xu C., Zhu L.-X., Sun D.-M., Yao H., Han D.-X. (2020). Regulatory mechanism of lncRNA NORAD on proliferation and invasion of ovarian cancer cells through miR-199a-3p. Eur. Rev. Med. Pharmacol. Sci..

[153] Liang H., Yu M., Yang R., Zhang L., Zhang L., Zhu D., Luo H., Hong Y., Yu T., Sun J. (2020). A PTAL-miR-101-FN1 Axis Promotes EMT and Invasion-Metastasis in Serous Ovarian Cancer. Mol. Ther. Oncolytics.

[154] Liang H., Yu T., Han Y., Jiang H., Wang C., You T., Zhao X., Shan H., Yang R., Yang L. (2018). LncRNA PTAR promotes EMT and invasion-metastasis in serous ovarian cancer by competitively binding miR-101-3p to regulate ZEB1 expression. Mol. Cancer.

[155] Yang X., Xin N., Qu H., Wei L., Han Z. (2020). Long noncoding RNA TUG1 facilitates cell ovarian cancer progression through targeting MiR-29b-3p/MDM2 axis. Anat. Rec..

[156] Li W., Ma S., Bai X., Pan W., Ai L., Tan W. (2020). Long noncoding RNA WDFY3-AS2 suppresses tumor progression by acting as a competing endogenous RNA of microRNA-18a in ovarian cancer. J. Cell. Physiol..

[157] Lin X., Spindler T.J., De Souza Fonseca M.A., Corona R.I., Seo J.H., Dezem F.S., Li L., Lee J.M., Long H.W., Sellers T.A. (2019). Super-Enhancer-Associated LncRNA UCA1 Interacts Directly with AMOT to Activate YAP Target Genes in Epithelial Ovarian Cancer. iScience.

[158] Qiu J., Lin X., Zheng T., Tang X., Hua K. (2018). Natural antisense transcript of hypoxia-inducible factor 1 regulates hypoxic cell apoptosis in epithelial ovarian cancer. Onco. Targets. Ther..

[159] Tang Z., Kang B., Li C., Chen T., Zhang Z. (2019). GEPIA2: An enhanced web server for large-scale expression profiling and interactive analysis. Nucleic Acids Res..

[160] Ghandi M., Huang F.W., Jané-Valbuena J., Kryukov G.V., Lo C.C., McDonald E.R., Barretina J., Gelfand E.T., Bielski C.M., Li H. (2019). Next-generation characterization of the Cancer Cell Line Encyclopedia. Nature.

[161] Li J., Han L., Roebuck P., Diao L., Liu L., Yuan Y., Weinstein J.N., Liang H. (2015). TANRIC: An interactive open platform to explore the function of lncRNAs in cancer. Cancer Res..

[162] Volders P.-J., Anckaert J., Verheggen K., Nuytens J., Martens L., Mestdagh P., Vandesompele J. (2019). LNCipedia 5: Towards a reference set of human long non-coding RNAs. Nucleic Acids Res..

[163] Fang S., Zhang L., Guo J., Niu Y., Wu Y., Li H., Zhao L., Li X., Teng X., Sun X. (2018). NONCODEV5: A comprehensive annotation database for long non-coding RNAs. Nucleic Acids Res..

[164] Hou M., Tang X., Tian F., Shi F., Liu F., Gao G. (2016). AnnoLnc: A web server for systematically annotating novel human lncRNAs. BMC Genom..

[165] Quek X.C., Thomson D.W., Maag J.L.V., Bartonicek N., Signal B., Clark M.B., Gloss B.S., Dinger M.E. (2015). lncRNAdb v2.0: Expanding the reference database for functional long noncoding RNAs. Nucleic Acids Res..

[166] Chakraborty S., Deb A., Maji R.K., Saha S., Ghosh Z. (2014). LncRBase: An enriched resource for lncRNA information. PLoS ONE.

[167] Lin Y., Liu T., Cui T., Wang Z., Zhang Y., Tan P., Huang Y., Yu J., Wang D. (2020). RNAInter in 2020: RNA interactome repository with increased coverage and annotation. Nucleic Acids Res..

[168] Li J.-H., Liu S., Zhou H., Qu L.-H., Yang J.-H. (2014). starBase v2.0: Decoding miRNA-ceRNA, miRNA-ncRNA and protein–RNA interaction networks from large-scale CLIP-Seq data. Nucleic Acids Res..

[169] Chou C.-H., Shrestha S., Yang C.-D., Chang N.-W., Lin Y.-L., Liao K.-W., Huang W.-C., Sun T.-H., Tu S.-J., Lee W.-H. (2018). miRTarBase update 2018: A resource for experimentally validated microRNA-target interactions. Nucleic Acids Res..

[170] Vafaee F., Krycer J.R., Ma X., Burykin T., James D.E., Kuncic Z. (2016). ORTI: An Open-access Repository of Transcriptional Interactions for interrogating mammalian gene expression data. PLoS ONE.

[171] Xiong Y., Wei Y., Gu Y., Zhang S., Lyu J., Zhang B., Chen C., Zhu J., Wang Y., Liu H. (2017). DiseaseMeth version 2.0: A major expansion and update of the human disease methylation database. Nucleic Acids Res..

[172] Zhi H., Li X., Wang P., Gao Y., Gao B., Zhou D., Zhang Y., Guo M., Yue M., Shen W. (2018). Lnc2Meth: A manually curated database of regulatory relationships between long non-coding RNAs and DNA methylation associated with human disease. Nucleic Acids Res..

[173] Cheng L., Wang P., Tian R., Wang S., Guo Q., Luo M., Zhou W., Liu G., Jiang H., Jiang Q. (2019). LncRNA2Target v2.0: A comprehensive database for target genes of lncRNAs in human and mouse. Nucleic Acids Res..

[174] Chen W., Zhang G., Li J., Zhang X., Huang S., Xiang S., Hu X., Liu C. (2019). CRISPRlnc: A manually curated database of validated sgRNAs for lncRNAs. Nucleic Acids Res..

[175] Hao Y., Zhang L., Niu Y., Cai T., Luo J., He S., Zhang B., Zhang D., Qin Y., Yang F. (2018). SmProt: A database of small proteins encoded by annotated coding and non-coding RNA loci. Brief. Bioinform..

[176] Zhao H., Shi J., Zhang Y., Xie A., Yu L., Zhang C., Lei J., Xu H., Leng Z., Li T. (2019). LncTarD: A manually-curated database of experimentally-supported functional lncRNA–target regulations in human diseases. Nucleic Acids Res..

[177] Bao Z., Yang Z., Huang Z., Zhou Y., Cui Q., Dong D. (2019). LncRNADisease 2.0: An updated database of long non-coding RNA-associated diseases. Nucleic Acids Res..

[178] Li Y., Li L., Wang Z., Pan T., Sahni N., Jin X., Wang G., Li J., Zheng X., Zhang Y. (2018). LncMAP: Pan-cancer atlas of long noncoding RNA-mediated transcriptional network perturbations. Nucleic Acids Res..

[179] Gao Y., Wang P., Wang Y., Ma X., Zhi H., Zhou D., Li X., Fang Y., Shen W., Xu Y. (2019). Lnc2Cancer v2.0: Updated database of experimentally supported long non-coding RNAs in human cancers. Nucleic Acids Res..

[180] Wang J., Zhang X., Chen W., Li J., Liu C. (2018). CRlncRNA: A manually curated database of cancer-related long non-coding RNAs with experimental proof of functions on clinicopathological and molecular features. BMC Med. Genom..

[181] Zhao Z., Zhou W., Han Y., Peng F., Wang R., Yu R., Wang C., Liang H., Guo Z., Gu Y. (2017). EMT-Regulome: A database for EMT-related regulatory interactions, motifs and network. Cell Death Dis..

[182] Wang C., Yang F., Chen T., Dong Q., Zhao Z., Liu Y., Chen B., Liang H., Yang H., Gu Y. (2019). RHPCG: A database of the Regulation of the Hippo Pathway in Cancer Genome. Database.

[183] Muppirala U.K., Honavar V.G., Dobbs D. (2011). Predicting RNA-Protein Interactions Using Only Sequence Information. BMC Bioinform..

[184] Suresh V., Liu L., Adjeroh D., Zhou X. (2015). RPI-Pred: Predicting ncRNA-protein interaction using sequence and structural information. Nucleic Acids Res..

[185] Lu Q., Ren S., Lu M., Zhang Y., Zhu D., Zhang X., Li T. (2013). Computational prediction of associations between long non-coding RNAs and proteins. BMC Genom..

[186] Agostini F., Zanzoni A., Klus P., Marchese D., Cirillo D., Tartaglia G.G. (2013). catRAPID omics: A web server for large-scale prediction of protein-RNA interactions. Bioinformatics.

[187] Tokar T., Pastrello C., Rossos A.E.M., Abovsky M., Hauschild A.-C., Tsay M., Lu R., Jurisica I. (2018). mirDIP 4.1—integrative database of human microRNA target predictions. Nucleic Acids Res..

[188] Fukunaga T., Iwakiri J., Ono Y., Hamada M. (2019). LncRRIsearch: A Web Server for lncRNA-RNA Interaction Prediction Integrated With Tissue-Specific Expression and Subcellular Localization Data. Front. Genet..

[189] He S., Zhang H., Liu H., Zhu H. (2015). LongTarget: A tool to predict lncRNA DNA-binding motifs and binding sites via Hoogsteen base-pairing analysis. Bioinformatics.

[190] Gruber A.R., Lorenz R., Bernhart S.H., Neubock R., Hofacker I.L. (2008). The Vienna RNA Websuite. Nucleic Acids Res..

[191] Lou U.K., Wong C.H., Chen Y. (2020). A simple and rapid colorimetric detection of serum lncRNA biomarkers for diagnosis of pancreatic cancer. RSC Adv..

[192] Islam M.N., Moriam S., Umer M., Phan H.-P., Salomon C., Kline R., Nguyen N.-T., Shiddiky M.J.A. (2018). Naked-eye and electrochemical detection of isothermally amplified HOTAIR long non-coding RNA. Analyst.

[193] Guo B., Wu S., Zhu X., Zhang L., Deng J., Li F., Wang Y., Zhang S., Wu R., Lu J. (2020). Micropeptide CIP2A-BP encoded by LINC00665 inhibits triple-negative breast cancer progression. EMBO J..

