# Correction: Salamini-Montemurri et al. The Challenges and Opportunities of LncRNAs in Ovarian Cancer Research and Clinical Use. *Cancers* 2020, *12*, 1020

**DOI:** 10.3390/cancers18142288

**Published:** 2026-07-16

**Authors:** Martín Salamini-Montemurri, Mónica Lamas-Maceiras, Aida Barreiro-Alonso, Ángel Vizoso-Vázquez, Esther Rodríguez-Belmonte, María Quindós-Varela, María Esperanza Cerdán

**Affiliations:** 1 EXPRELA Group, Centro de Investigacións Científicas Avanzadas (CICA), Departamento de Bioloxía, Facultade de Ciencias, INIBIC-Universidade da Coruña, Campus de A Coruña, 15071 A Coruña, Spain; martin.salamini.montemurri@udc.es (M.S.-M.); monica.lamas@udc.es (M.L.-M.); aida.barreiro@udc.es (A.B.-A.); a.vizoso@udc.es (Á.V.-V.); esther.belmonte@udc.es (E.R.-B.); 2 Translational Cancer Research Group, Instituto de Investigación Biomédica de A Coruña (INIBIC), Carretera del Pasaje s/n, 15006 A Coruña, Spain; maria.quindos.varela@sergas.es

Following publication, the Editorial Office have become aware that a number of original articles cited in [1] were retracted by their respective journals/publishers. The authors stated that cited papers were retracted after publication of [1] and that that does not affect the scientific conclusions.

The following corrections have been made, and the corresponding references and related content have been removed [16,50,52,59,67,78,95,99,115,116,126,128,143,144,155] to ensure scientific accuracy. Specifically:

- From Tables 1–5, affected examples of lncRNAs with the corresponding reference have been removed and the remaining references have been re-numbered.
- From Figure 3, the affected example of lncRNAs “HAL” has been removed.
- From sections “Clinical Relevance of LncRNA in OC: Diagnosis, Prognosis and Treatment Resistance”, “LncRNAs Implicated in OC Development and Progression” and “Regulatory Molecular Mechanisms of LncRNAs in OC”, affected examples of lncRNAs with the corresponding reference have been removed and the remaining references have been re-numbered.

Retracted references and corresponding information have been also removed from Supplementary Table S1 in the section “Supplemenatry materials”.

With this correction, the order of the remaining references has been adjusted accordingly. The authors state that the scientific conclusions are unaffected. This correction was approved by the Academic Editor. The original publication has also been updated.
